# Rising incidence of carbapenem-resistant *Citrobacter* spp. in a German tertiary-care hospital: epidemiology, clinical impact, and the role of the hospital wastewater system—findings from a six-year molecular study

**DOI:** 10.1128/spectrum.02670-25

**Published:** 2026-01-22

**Authors:** Helena Tsakmaklis, Niklas M. Weidner, Kaan Kocer, Rouven Behnisch, Dirk Jäger, Patrick Michl, Markus Mieth, Elena Busch, Christian Brandt, Alexander H. Dalpke, Dennis Nurjadi, Sebastien Boutin, Isabel Späth

**Affiliations:** 1 Department of Infectious Diseases, Medical Microbiology and Hygiene, Heidelberg University, Medical Faculty Heidelberg 64338, Heidelberg, Germany; 2 Institute of Medical Microbiology, University Hospital Schleswig-Holstein Campus Lübeck and University of Lübeck, Lübeck, Germany; 3 German Center for Infection Research (DZIF), Partner Site Hamburg-Lübeck-Borstel-Riems, Lübeck, Germany; 4 Institute of Medical Biometry (IMBI), Heidelberg University 7891, Heidelberg, Germany; 5 National Center for Tumor Diseases, Heidelberg University, Medical Faculty Heidelberg, University Hospital Heidelberg 9144, Heidelberg, Germany; 6 Department of Gastroenterology, Hepatology and Infectious Diseases, Heidelberg University, Medical Faculty Heidelberg, University Hospital Heidelberg 27178, Heidelberg, Germany; 7 Department of General, Visceral and Transplant Surgery, Heidelberg University, Medical Faculty Heidelberg, University Hospital Heidelberg 27178, Heidelberg, Germany; 8 Department of Hematology, Oncology and Rheumatology, Heidelberg University, Medical Faculty Heidelberg, University Hospital Heidelberg 236491 , Heidelberg, Germany; 9 Translational Lung Research Center (TLRC), German Center for Lung Research (DZL), Heidelberg, Germany; 10 Airway Research Center North (ARCN), German Center for Lung Research (DZL), Lübeck, Germany; Sacramento County Public Health Laboratory, Sacramento, California, USA

**Keywords:** carbapenem-resistant *Enterobacterales*, *Citrobacter* spp., incidence rate, hospital wastewater system

## Abstract

In recent years, a threatening rise in carbapenem-resistant *Citrobacter* spp. (CRC) has been observed in various global regions. Sewage systems have repeatedly been suggested as reservoirs. At our tertiary-care hospital in Germany, CRC have become the most frequently detected species among the carbapenem-resistant *Enterobacterales* (CRE) over the past six years. This study aimed to analyze CRC’s site-specific epidemiological development, assess their clinical significance, and elucidate the potential role of the hospital wastewater system in the observed increase in CRC. A retrospective epidemiological investigation was conducted using Poisson models. Clinical baseline data of affected patients were evaluated. Whole-genome sequencing and a subcluster analysis of 138 CRC patient isolates were performed. The wastewater systems of three clinical wards located in two separate hospital buildings were examined. The annual proportion of CRC in CRE increased from 9.2% in 2019 to 37.8% in 2024 (*P* < 0.001). 27.5% of isolates were detected in clinical specimens, most frequently in urine samples (52.6%). There were no cases of bacteremia. We identified 26 MLST types and 22 subclusters. Ninety-three percent of isolates had one (77.5%), two (14.7%), or three (0.8%) carbapenemases. Wastewater analysis identified 27 of 37 (73.0%) environmental CRC isolates as genetically consistent with patient-derived strains. Our findings reveal a newly emerging complexity, characterized by the spread of various genetically distinct CRC across several departments of our hospital. While CRC colonization increased, the frequency and severity of infections remained moderate. The hospital wastewater system represents a key factor in CRC’s dissemination.

**IMPORTANCE:**

In this study, we analyzed the significant increase in CRC strains at our tertiary-care hospital over the past six years. Our findings revealed that, unlike classical outbreak scenarios — typically characterized by the clonal expansion of one strain within a single ward or unit — the rising incidence of CRC was driven by the dissemination of numerous genetically diverse strains across multiple departments. The hospital sewage system was identified as a reservoir and plays an important role in the rapid proliferation and dissemination of CRC. The high complexity of the situation presents a new challenge, as transmission pathways are now difficult to trace, hindering effective prevention. Remarkably, the majority of patients affected by CRC were merely colonized, and severe infections were not observed. It remains to be determined whether this pattern reflects a site-specific phenomenon or indicates a broader trend on a European or even global scale.

## INTRODUCTION

*Citrobacter* spp. are gram-negative, facultative anaerobic bacteria that can be found in water, soil, and food, as well as in the intestinal tracts of animals and humans (1). They are opportunistic pathogens causing a broad spectrum of primarily hospital-acquired infections, such as urinary tract infections, bloodstream infections, pneumonia, and meningitis, especially in patients with severe underlying conditions (1, 2).

Recently, carbapenem-resistant *Citrobacter* spp. (CRC) have emerged as important healthcare-associated pathogens, predominantly in China, but also in the United States and Europe (3–8). Furthermore, a growing number of CRC-related outbreaks have been reported in recent years, particularly in hemato-oncology departments (9–11). The wastewater system has repeatedly been identified as a reservoir in this context (10, 12, 13).

In our tertiary-care hospital in Germany, we observed a significant increase in CRC detections over the past six years. In order to analyze the epidemiological situation in our setting, we conducted a retrospective epidemiological investigation of the incidence of CRC, comparing it to other common carbapenem-resistant *Enterobacterales* (CRE), such as *Klebsiella pneumoniae*, *Escherichia coli*, and *Enterobacter cloacae* complex. To assess the clinical impact of the marked increase in CRC, basic clinical characteristics of the affected patients, such as sex, age, medical department, microbiological findings, and incidence of bacteremia, were evaluated. To gain a more detailed understanding of the transmission mechanisms, molecular typing of 138 CRC patient isolates was performed using whole-genome sequencing (WGS) and a subsequent cluster analysis. Moreover, the wastewater systems of all rooms on three clinical wards, located in two different hospital buildings, were sampled, and genetic relatedness between environmental and patient isolates was analyzed.

## MATERIALS AND METHODS

### Setting, patients, collection of isolates, routine screening, and infection control measures

#### Setting

Our study was conducted at a 2,600-bed tertiary-care hospital in Germany, which treats about 85,500 inpatients annually.

#### Patients

Only inpatients who spent at least one night in the hospital were included. Colonized patients were defined as those testing positive with CRC detected from rectal swabs. A possible infection was assumed if CRC were found in clinical material. A relevant infection was identified if blood cultures or cerebrospinal fluid tested positive for CRC. A hospital-acquired infection or colonization was considered to have occurred when CRC isolates assigned to a specific subcluster were identified.

#### Collection of clinical isolates

We included the following carbapenem-resistant bacteria in our analysis: *Citrobacter* spp., *Enterobacter cloacae* complex, *Escherichia coli*, and *Klebsiella pneumoniae*. A total of 560 isolates were obtained between 2019 and 2024, both from rectal swabs as part of the routine multidrug-resistant organism (MDRO) admission screening and from clinical microbiological samples of inpatients. We had to exclude 31 (5.5%) isolates detected in clinical materials of outpatients and 19 (3.4%) isolates due to methodical inconsistencies.

#### Collection of environmental isolates

Water samples were collected twice from all patient bathrooms of three different wards: in December 2021 and 2024 in ward X1, located in the surgery building; in February and April 2023 in ward X2; and in February and December 2023 in ward X3. The last two wards are both located in the internal medicine building. We collected 50 mL of wastewater from the drainpipe of each toilet and the water trap of the showers (where available, ward X1 has only one shower in a separate bathroom, while all rooms in wards X2 and X3 are equipped with showers). We included only CRC isolates for further analysis. The floor plans of the investigated wards, shown in Fig. 5, which indicate the recovery sites of environmental isolates, are created using the Roomsketcher app.

#### Routine screening

The MDRO admission screening was performed as part of the hospital’s infection control strategy and consisted of a nasal and a rectal swab. These were taken from “patients at risk,” as defined by the Department of Microbiology and Hygiene, according to the recommendations of the Commission of Hospital Hygiene and Infection Prevention (KRINKO) at the Robert Koch Institute in Germany (14, 15). In the hemato-oncology wards and intensive care units, all patients were examined on admission and then underwent weekly screening.

#### Infection control measures

Contact precautions, including single-room isolation and wearing of personal protective equipment before contact with patient or the patient environment, were implemented immediately after the detection of CRE in a microbiological sample.

### Laboratory methods

#### Microbiological examination of patient samples

Samples were inoculated on chromID ESBL agar (bioMérieux, Germany), and the plates were incubated for exactly 24 h at 36 ± 1°C using a total lab automation (BD Kiestra) that included digital imaging. Species identification was performed via matrix-assisted laser desorption ionization–time of flight (MALDI-TOF) (MBT Sirius one, Bruker), and antimicrobial susceptibility testing (AST) was performed using VITEK 2 (bioMérieux, Germany). The results were interpreted according to the current European Committee on Antimicrobial Susceptibility Testing (EUCAST) clinical breakpoints version for the respective year. Carbapenem-resistant isolates were cryopreserved at −20°C for molecular typing.

#### Microbiological examination of environmental samples

After an in-house validation of different sample volumes, 10 µL was selected as the optimal volume, providing sufficient sensitivity while minimizing overgrowth. Ten microliters of each water sample were first plated onto a carbapenem-containing selective agar (ChromID CARBA SMART, biomérieux, Germany), and the plates were incubated for 24 h at 36 ± 1°C. Cultured isolates were identified via MALDI-TOF. WGS was performed on all detected *Citrobacter* spp. isolates.

#### DNA extraction and whole-genome sequencing

WGS was performed on all carbapenem-resistant isolates. Bacterial genomic DNA was extracted from the overnight cultures with the DNeasy Blood and Tissue Kit (Qiagen GmbH, Germany), following the manufacturer’s protocol. DNA libraries were created using the Nextera DNA Flex Library Prep Kit (Illumina, USA) and sequenced on a MiSeq or NextSeq 1000 (2 × 300 bp paired-end) at the Department of Infectious Disease, Medical Microbiology and Hygiene, Heidelberg. Quality filtering of raw sequences was performed using fastp (v 0.23.4 with parameters -q 30 -l 45) (16) and assembly with SPAdes (v 3.15.5 with parameters --careful and --only-assembler) (17). Curation of the draft genomes was performed by removing contigs with a length <500 bp or coverage < 10×. The quality of the final draft was assessed using Quast (v5.2.0) (16, 18). Draft genomes were screened against RefSeq reference genomes using Mash (v2.3) with a maximum cut-off distance of 0.05 for species determination (19). Carbapenemase genes were identified using Abricate (v1.0.1) with a custom database containing carbapenemase genes based on Resfinder and CARD (20, 21). Carbapenemase-containing contigs were extracted from the draft genomes and compared against the PLSDB database using the BLAST+ suite (File S1) (22, 23).

#### Phylogenetic analysis and cluster detection

Typing was conducted using multilocus sequence typing (MLST) and by assigning isolates to variable-length k-mer clusters (VLKC) with PopPUNK (24), using a Bayesian Gaussian Mixture Model. Draft genomes identified as *Citrobacter freundii* were first divided into VLKC. For subsequent single nucleotide polymorphism (SNP)-based analysis of isolates with identical VLKC, draft genomes were aligned to the reference genome and core SNP genome constructed using Snippy (v4.6.0), recombinations masked with Gubbins (v3.3.1) (25), and SNP matrices calculated with snp-dists (v0.8.2). Each VLKC with >5 isolates was analyzed separately with a representative draft genome of the respective VLKC. For VLKC with 2–5 isolates and non-*freundii* species, a SNP analysis was conducted in a species-wide alignment to the RefSeq reference genomes of *Citrobacter freundii*, *Citrobacter portucalensis*, or *Citrobacter braakii*, respectively.

Clusters were detected using hierarchical cluster analysis with complete linkage and a cutoff of 20 SNPs. This threshold was derived from the authors’ experience in outbreak investigations, accounting for higher variability of long-term outbreaks from an environmental source. Isolates were classified based on the size of the associated cluster: singleton (no cluster), cluster with 2–3 isolates, or cluster with >3 isolates.

### Statistical analysis

The normally distributed data and results were presented as means and standard deviations (SDs), while categorical data were summarized with frequencies and percentages. Statistical significance was determined using the Chi-square (χ^2^) test. The incidence rate was defined as the number of hospitalized patients affected by CRE per 100,000 patient days. The factors expressing the increase/decrease in incidence rates were calculated using Poisson models and presented together with the 95% confidence interval (CI). All statistical analyses were performed using R Core Team, version 4.3.2 (26).

## RESULTS

### Epidemiology

**Fig 1:**
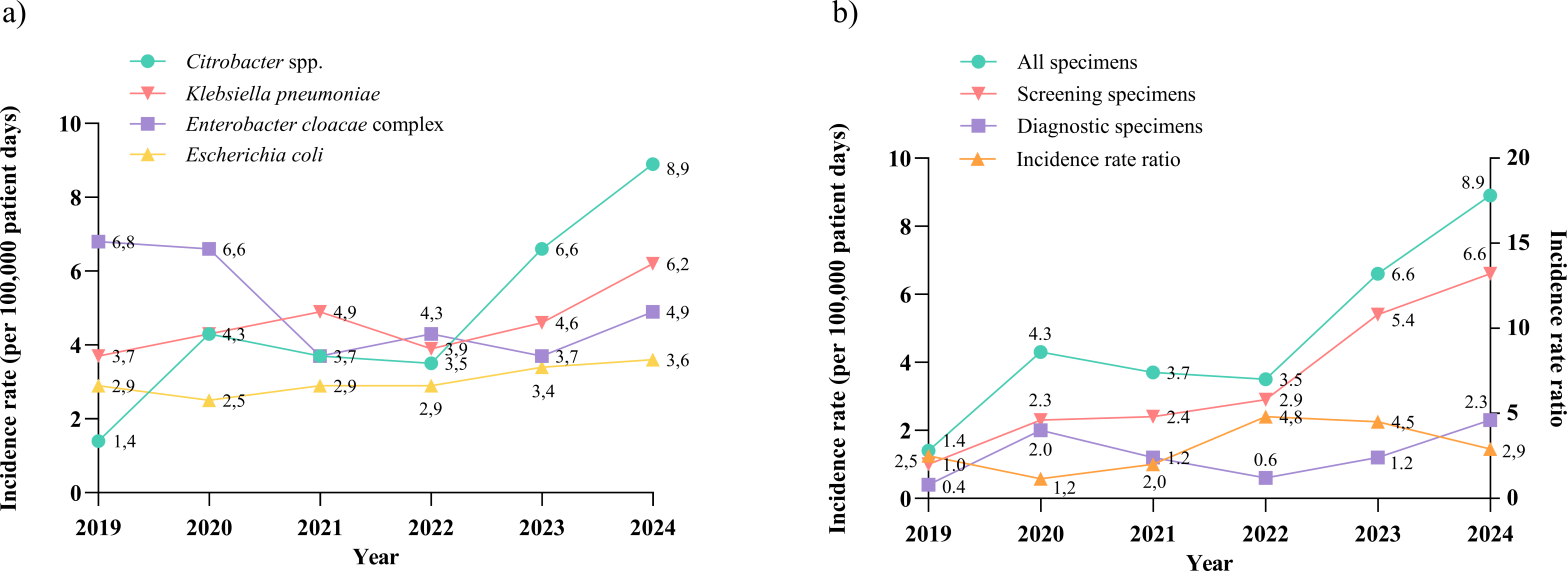
(**a**) Annual incidence rates of carbapenem-resistant *Citrobacter* spp., *Enterobacter cloacae* complex, *Escherichia coli,* and *Klebsiella pneumoniae* from 2019 to 2024. (**b**) Left axis: Annual incidence rates of patients with carbapenem-resistant *Citrobacter* spp. detected in screening specimens and in diagnostic specimens compared to the overall incidence rates from 2019 to 2024. Right axis: The incidence rate ratio of screening specimens to diagnostic specimens.

A total of 560 CRE patient isolates were detected at our facility between January 2019 and December 2024, of which 510 isolates were included in this study: 138 *Citrobacter* spp., 148 *Enterobacter cloacae* complex, 135 *Klebsiella pneumoniae*, and 89 *Escherichia coli* strains. The annual proportion of CRC in the total number of CRE increased from 9.2% in 2019 to 37.8% in 2024 (*P* < 0.001). We calculated the annual incidence rates (IR) for each species over the six-year period (Fig. 1a). Based on the Poisson model, the overall IR for CRC increased annually by a factor of 1.33 (95% CI, 1.20–1.46), corresponding to a relative increase of 33% (File S2, Section A). For carbapenem-resistant *Enterobacter cloacae* complex, *Escherichia coli*, and *Klebsiella pneumoniae*, the respective factors were 0.9, 1.06, and 1.08 (corresponding to a decrease of 10% and increases of 6% and 8%), with only CRC showing a significant increase (*P* < 0.001). Remarkably, the IR of *Enterobacter cloacae* complex was higher in 2019 and 2020 than in the following years. There was a major outbreak of *Enterobacter cloacae* complex in our hospital over this period, which was contained by special infection control measures in 2020 (27). After exclusion of the outbreak, analysis of the IR of *Enterobacter cloacae* complex demonstrated a factor of 1.06 and a relative annual increase of 6% (File S2, Section B).

To ensure that fluctuations in annual screening rates did not significantly influence the increase in CRE detections over the six-year period, we additionally calculated the IR taking into account the screening volume (File S2, Section C). However, the relative increase in IR remained virtually unchanged at 33%, 7%, 6%, and 8% for *Citrobacter* spp., *Enterobacter cloacae* complex, *Escherichia coli*, and *Klebsiella pneumoniae*, respectively.

### Clinical data

64.5% of patients affected by CRC were male. The majority of the isolates (43.5%) were found in patients aged between 61 and 80 years. The average age of colonized or infected patients was 60.6 years (SD 17.7 years).

**Fig 2:**
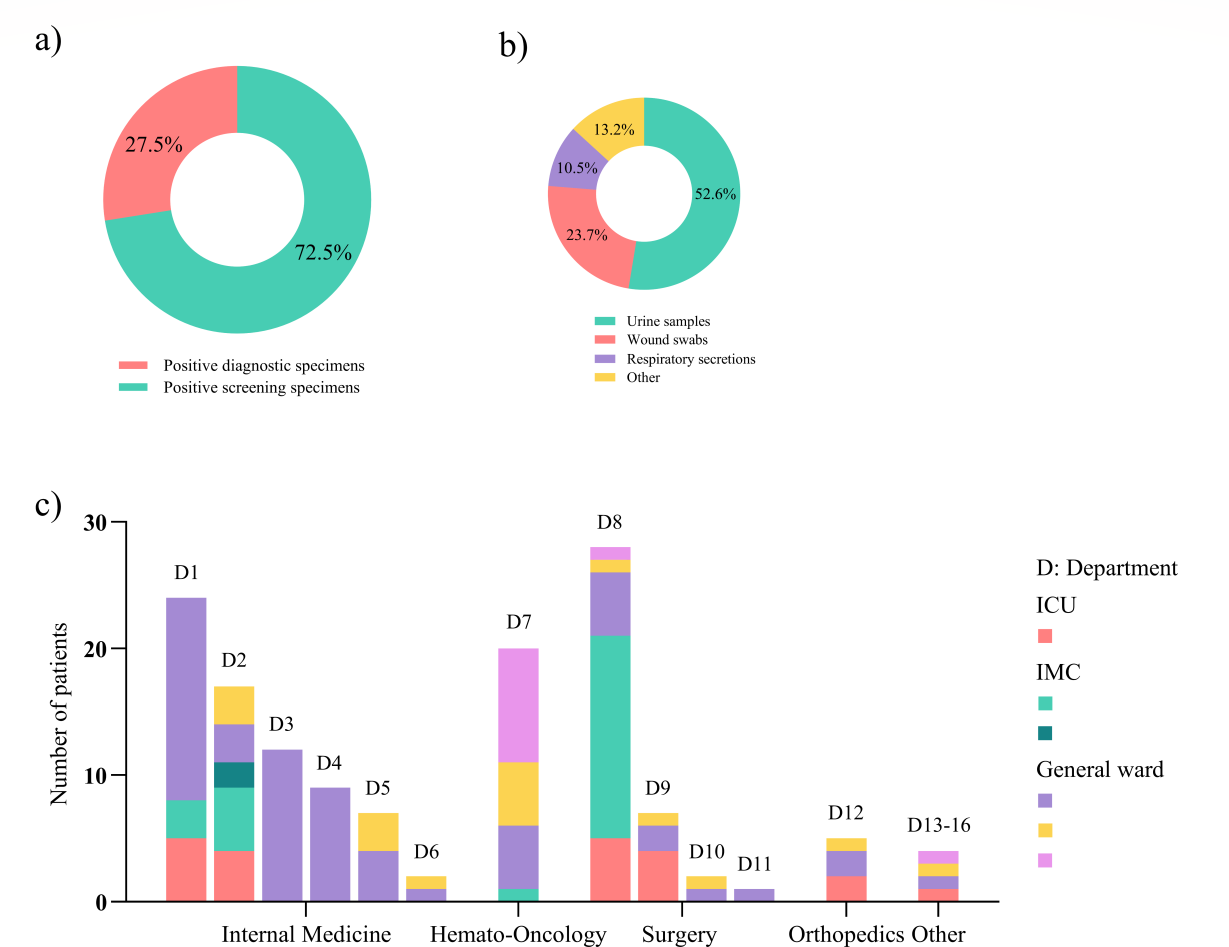
(**a**) Proportion of CRC isolates obtained from screening specimens and from diagnostic specimens. (**b**) Diagnostic specimens in which CRC were found. (**c**) Overview of the departments and the number of involved ICU, IMC, and general wards, where patients with CRC were treated at the time of initial detection.

One hundred (72.5%) isolates were obtained from screening specimens and 38 (27.5%) from diagnostic specimens (Fig. 2a). Over the six-year period, the IR of patients who tested positive in diagnostic specimens remained relatively stable. However, the IR of patients who tested positive in screening specimens showed a significant increase (factor 1.4, 95% CI, 1.20–1.63, relative increase of 40%, *P* < 0.001), which was consistent with the overall IR of CRC (Fig. 1b), suggesting that the increase in overall CRC detections was mainly due to increased colonizations. This hypothesis is supported by the incidence rate ratio (IRR) of screening to diagnostic specimens, which increased from 2020 to 2022 and showed only a slight decrease in 2023 (Fig. 1b). However, the decline in IRR in 2024 needs further monitoring. Most isolates in diagnostic specimens were found in urine samples (20/38, 52.6%), followed by wound swabs (9/38, 23.7%), respiratory secretions (4/38, 10.5%), and other materials (Fig. 2b). No CRC were detected in blood cultures or cerebrospinal fluid.

Figure 2c displays the distribution of departments and wards in which patients were treated at the time of initial CRC detection. The majority of patients who tested positive had been treated in various departments of internal medicine (including hemato-oncology) or surgery. The ratio of colonized patients to patients who tested positive in diagnostic specimens was approximately three to one, both in general wards (72.5%/27.5%) and ICU/IMC (72.3%/27.7%). All hemato-oncology patients were only colonized.

### Molecular analysis

#### Phylogeny, MLST, VLKC, subcluster, and carbapenemases

Out of 138 CRC isolates, 129 (93.5%) were identified as *Citrobacter freundii*, 4 (2.9%) as *Citrobacter braakii*, 4 (2.9%) as *Citrobacter portucalensis*, and 1 (0.7%) as *Citrobacter pasteurii* by sequencing.

**Fig 3:**
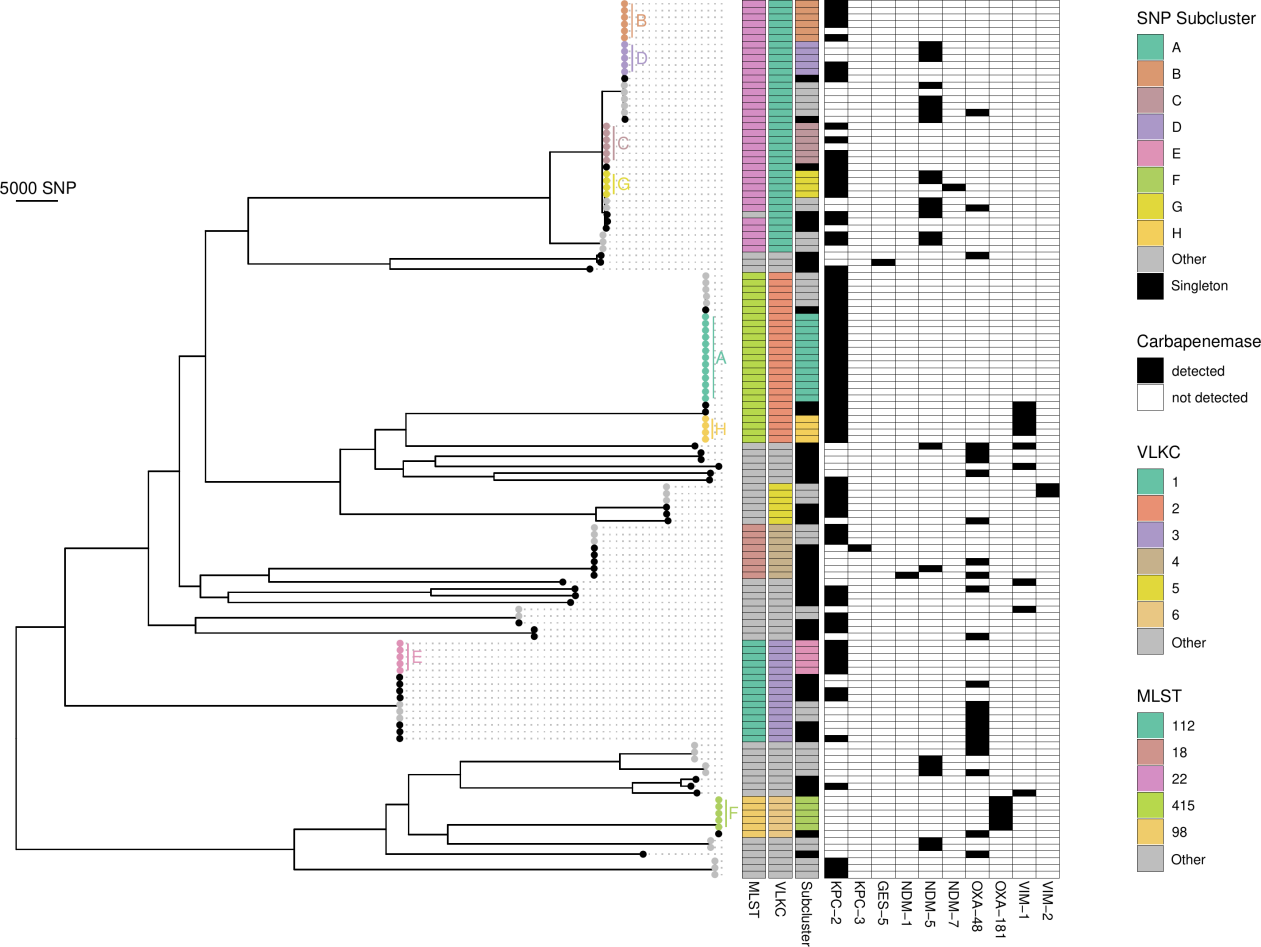
Phylogenetic tree of carbapenem-resistant *Citrobacter freundii* patient isolates, constructed using Gubbins, annotated with assigned MLST, VLKC, SNP-based subcluster, and detected carbapenemases. MLST, multilocus sequence typing; SNP, single nucleotide polymorphism; VLKC, variable-length k-mer cluster.

Further phylogenetic analysis of carbapenem-resistant *Citrobacter freundii* isolates was performed by MLST and VLKC assignment, along with SNP comparisons of isolates within identical VLKC (Fig. 3). Transmission events between isolates assigned to different VLKC can be ruled out since they are genetically sufficiently distinct (24, 28). Carbapenem-resistant *Citrobacter freundii* isolates were grouped into subclusters if they were less than 20 SNPs apart. Forty-five (34.9%) of the 129 carbapenem-resistant *Citrobacter freundii* isolates were classified as singleton without a subcluster assignment, and 84 isolates (65.1%) were assigned to different subclusters (Table 1): 36 isolates belonged to 14 small subclusters (2–3 isolates; subclusters a–n), and 48 isolates belonged to eight larger subclusters (>3 isolates; subclusters A–H). A total of 26 MLST types were identified, with the most common being ST22 (*n* = 36, 27.9%), ST415 (*n* = 25, 19.4%), ST112 (*n* = 15, 11.6%), ST18 (*n* = 8, 6.2%), and ST98 (*n* = 6, 4.7%). The assigned VLKC was largely consistent with MLST. No transmission events were detected in the non-*freundii* species by SNP-based typing.

**TABLE 1:** MLST type, VLKC, and the corresponding number of *Citrobacter freundii* isolates, which were assigned to small subclusters (a–n) or to large subclusters (A–H)^*a*^

MLST	VLKC	Total no. of isolates	Total no. of isolates (%)	No. of isolates assigned to subclusters (a–n)	No. of isolates assigned to subclusters (A–H)
ST22	1	31	86.1	3 (a), 2 (b), 2 (c), 3 (d)	6 (B), 6 (C), 5 (D), 4 (G)
ST415	2	22	88.0	3 (g), 2 (h)	13 (A), 4 (H)
ST112	3	8	53.3	3 (i)	5 (E)
ST18	4	3	37.5	3 (j)	0
ST98	6	5	83.4	0	5 (F)
other		15	38.5	2 (e), 2 (f), 3 (k), 2 (l), 3 (m), 3 (n)	0
all		84	65.1	36	48

^*a*^ MLST, multilocus sequence typing; no., number; VLKC, variable-length k–mer cluster.

At least one carbapenemase gene was identified in 120 of 129 (93%) carbapenem-resistant *Citrobacter freundii* isolates. Two carbapenemase genes were found in 19 isolates (14.7%), and one isolate even carried three. The most common carbapenemase gene was *bla*_KPC-2_ (*n* = 74, 57.4%), followed by *bla*_OXA-48_ (*n* = 25, 19.4%) and *bla*_NDM-5_ (*n* = 22, 17.0%). Notably, the proportion of isolates with no carbapenemase genes was higher in other species: 34.2%, 20.0%, and 23.0% in *Enterobacter cloacae* complex, *Escherichia coli*, and *Klebsiella pneumoniae*, respectively.

### Subcluster analysis

**Fig 4:**
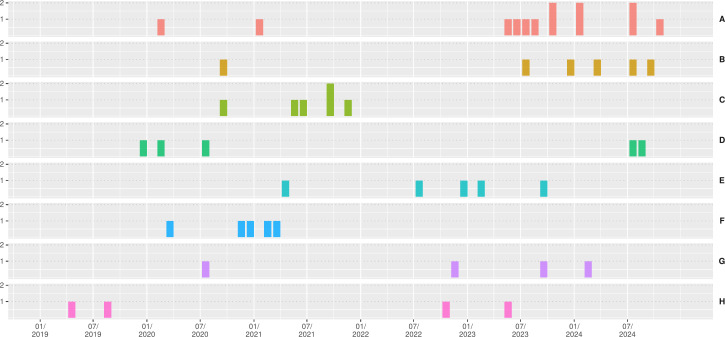
Histogram of the first detection of the respective isolates belonging to subclusters with >3 isolates, grouped by month for each subcluster. The number of isolates per month and subcluster is indicated on the left, and subcluster designation on the right. The year is shown on the x-axis.

Figure 4 shows the different isolates of subclusters A to H according to the time of first detection. Several detections occurred months to years apart, suggesting that the corresponding patients had no spatiotemporal overlap. For all large subclusters, patient records were reviewed to assess potential contact during inpatient or outpatient care. Overall, no documented epidemiologic link to other affected patients could be identified in 35 of 47 (74.5%) cases (File S3). Given the high genetic similarity of the isolates within each subcluster, independent acquisition of resistance determinants in these isolates appeared highly unlikely. We hypothesized that in cases of hospital-acquired infection/colonization without detectable epidemiological link to other affected patients, transmission from the environment, particularly from the sewage system, which provides especially favorable conditions for bacterial survival, was the most likely explanation for the acquisition of CRC.

### Wastewater investigation

**Fig 5:**
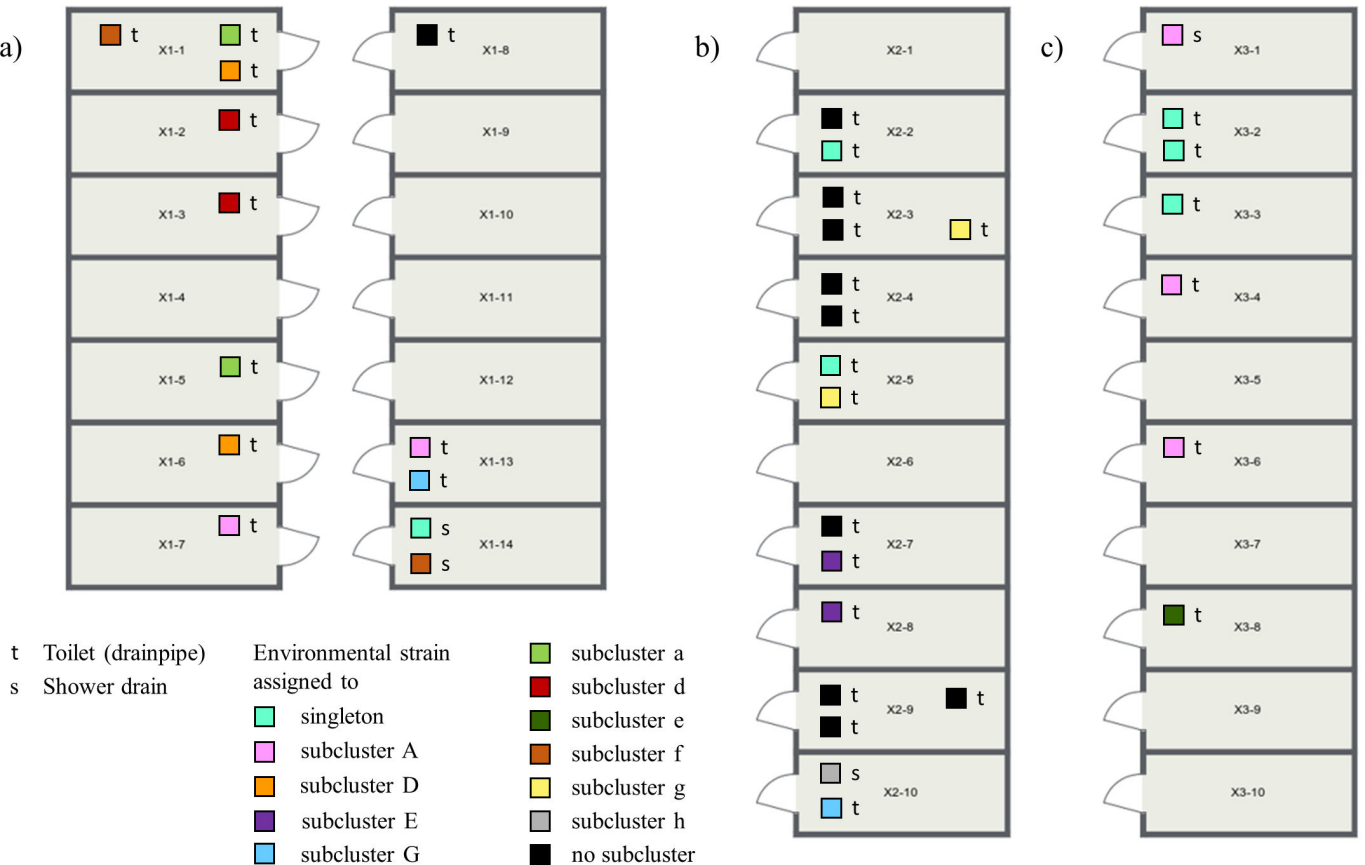
Floor plan of wards (**a**) X1, (**b**) X2, and (**c**) X3 with CRC isolates detected in toilet drainpipes or shower drains. Environmental isolates with genetic relatedness to different patient-derived strains are marked in colors, while isolates without subcluster affiliation are marked in black.

**Fig 6:**
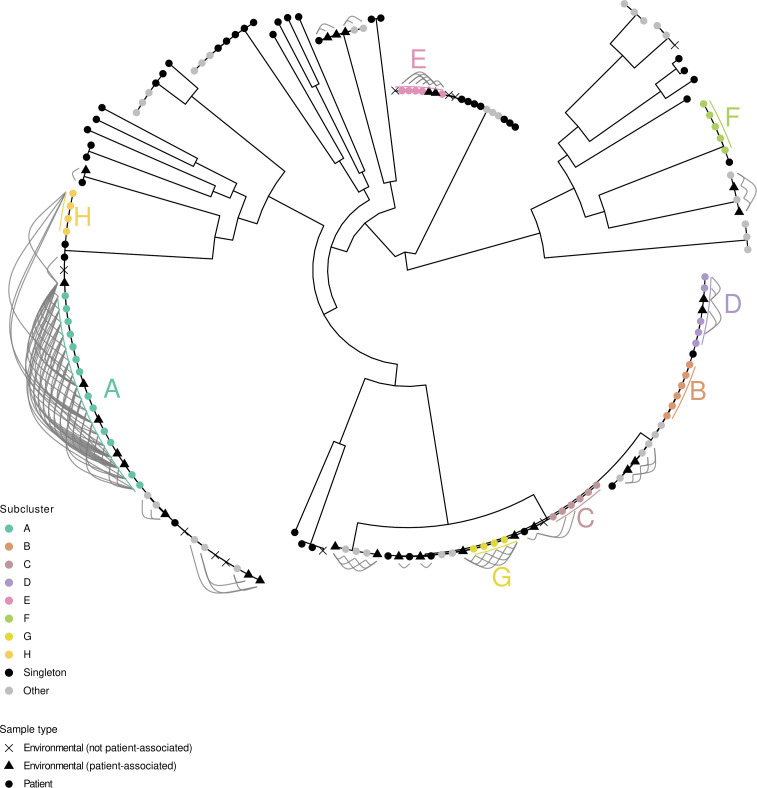
Phylogenetic tree of carbapenem-resistant *Citrobacter freundii* patient and environmental isolates, constructed using Gubbins. A cluster analysis was conducted solely on patient isolates. SNP-based distances of ≤20 SNPs between patient and environmental isolates are represented by connecting links.

Several patients with CRC were identified while they stayed in the surgical ward X1 (Department D8) or the internal medicine wards X2 (Department D3) and X3 (Department D1). Therefore, a wastewater analysis was carried out on these wards. A total of 53 environmental CRC isolates were obtained, of which 16 duplicates were excluded after genotyping. All of the remaining 37 genetically distinct isolates were identified as *C. freundii*. Twenty-seven of 37 (73.0%) environmental isolates showed genetic relatedness to patient-derived strains: 21 of these belonged to subcluster strains, and six to single-patient strains. Figure 5 displays the floor plan of the investigated wards and the recovery sites of the 37 isolates. The genetic relatedness of patient and environmental isolates is presented in Fig. 6. Pairwise distance distributions are provided in File S4.

Ward X2 was selected to illustrate potential transmission pathways (File S5). The isolates from the drainpipe of the toilets in rooms 3 and 5 belonged to subcluster g. Patients 1g and 2g, who consecutively occupied room 3, had never met during an inpatient or outpatient stay. Therefore, patient 2g might have acquired the subcluster isolate persisting in the toilet drain of room 3. Patient 3g was treated in room 5 after patients 1g and 2g, who had previously tested positive, had resided in room 5. Transmission via the toilet drain could also have occurred in this case. However, patients 2g and 3g were treated simultaneously on ward X2, so patient-to-patient transmission could not be ruled out.

We obtained subcluster E isolates in the toilet drains of rooms 7 and 8. Patient 1E had stayed in rooms 4, 5, and 6. More than 12 months later, patient 2E also stayed in these rooms and may have acquired the subcluster isolate via the sanitary facilities, although it was no longer detected in the wastewater analysis. Patient 3E tested positive following admission to room 8, five months after the positive patient 2E had been residing in that room. Two months later, patient 4E, also staying in room 8, tested positive. However, at this time, patient 3E was still residing in the neighboring room 7, so patient-to-patient transmission might have occurred. After another seven months, an additional patient (5E) showed the isolate after staying in room 8.

The isolate obtained in the shower drain of room 10 belonged to subcluster h. Patient 1h stayed in room 10 and tested positive. Both transmission from the patient to the environment and transmission from the environment to the patient seemed to be possible. Patient 2h had not been treated on ward X2, and there was no obvious epidemiological link between the two patients. In this case, the transmission route remained unclear.

In addition, we detected an isolate in room 10 that belonged to subcluster G. Interestingly, none of the four corresponding patients had ever been accommodated on ward X2.

In summary, 40 patients were found to be colonized by or infected with subcluster isolates detected in the sewage systems of wards X1, X2, and X3 (subclusters A, D, G, E, a, d, e, f, g, h). Concordance between patient isolates and the isolates detected in the wastewater analysis of the rooms they respectively occupied was proven in 18 of these cases (45.0%) (File S6). Some colonized or infected patients had never been treated on wards X1, X2, or X3, or had received treatment in one of these three wards as well as others. Due to the absence of wastewater testing data for these other areas, transmission routes could not be comprehensively determined.

## DISCUSSION

Hospital-acquired infections with difficult-to-treat, CRE pose an increasing threat to patients (29). *Klebsiella pneumoniae* and *Escherichia coli* are known to be the predominant species detected in the hospital setting (30, 31). However, from 2019 to 2024, we observed a pronounced rise in the occurrence of CRC in our tertiary-care hospital. The proportion of CRC among CRE increased significantly from 9.2% in 2019 to 37.8% in 2024, making CRC the most frequently detected species within the *Enterobacterales* group. In Spain, Arana et al. observed only a slight increase in CRC from 1.3% to 1.5% in a national surveillance program from 2013 to 2015 (8). However, in a German surveillance study from 2017 to 2019, which included 61 participating hospitals, a much greater increase from 10% to 14% was observed (7). Some authors describe the epidemiological development of CRC by indicating the rates of resistant isolates among the total number of *Citrobacter* species. In a US-based study, Babiker et al. reported an increase from 4% to 10% from 2000 to 2018 (6), and Ju et al. reported an overall rate of 7.9% between 2016 and 2023, involving six tertiary-care hospitals in China (4). This prompts the question of whether our findings represent a site-specific phenomenon or indicate a more widespread, general trend. The reports by Yao et al. and Ju et al., which included data from a large number of hospitals, suggest a broader trend. However, further studies are needed to confirm this development.

In this study, the overall proportion of positive tested diagnostic specimens was 27.5%. Interestingly, over the six-year period, the incidence rate of positive detections in screening specimens increased significantly, whereas the rate of positive detections in diagnostic specimens remained relatively stable, with a slight decrease from 2020 to 2022, followed by a resurgence from 2022 to 2024. These findings may suggest that the increase in overall detections of CRC is mainly due to an increase in colonizations. Yet, the rising detections in clinical specimens in 2024 require further monitoring. Most positive clinical samples were urine samples, followed by wound swabs. There were no cases of bacteremia in six years, and no infections occurred in the hemato-oncology department. These observations contrast with reports of severe illness caused by CRC in past years (1, 2, 32, 33) and with previous outbreak reports in hemato-oncology showing potentially fatal septic presentations (9, 10, 12), indicating a lower overall infection severity in our setting. Our data suggest that an increased acquisition of resistance determinants in CRC is not associated with increased virulence. Whether the virulence of strains is influenced by MLST type remains unclear, due to a lack of sufficient supporting data.

In the phylogenetic analysis, carbapenem-resistant *C. freundii* was the predominant species, at 93.5%. This result is consistent with previous reports (3, 4, 6, 7). 93.0% of carbapenem-resistant *C. freundii* isolates were carbapenemase producers, indicating that carbapenem resistance was predominantly due to the acquisition of carbapenemases. Interestingly, we confirmed the results of Yao et al., observing a higher proportion of carbapenemase producers in *Citrobacter* spp. than in other *Enterobacterales* (7).

However, in our study, the number of isolates carrying two carbapenemases exceeded that reported by other authors: 14.7% compared to 4.0%, 3.7%, and 0% in the respective investigations by Yao et al., Zhao et al., and Babiker et al. (3, 6, 7). Furthermore, we detected one isolate co-harboring the carbapenemase genes *bla*_OXA-48_, *bla*_NDM-5_, and *bla*_VIM-1_.

We identified a total of 26 different MLST types, with ST22 and ST415 being the most common. We also found that 65.1% of all isolates belonged to one of the 22 total subclusters. The large number of MLST types and subclusters, indicating a high genetic diversity, might be explained by prior reports of carbapenemase genes of *Citrobacter* spp. being predominantly localized on plasmids, which are transferable by conjugation (3, 7).

The substantial increase in CRC appears to be due to both pronounced horizontal gene transfer and clonal transmission. We hypothesize that the hospital wastewater system constitutes a key contributing factor to this development. Ju et al. identified the hospital wastewater system as the most important reservoir for CRC compared to human and animal sources. They observed a significant increase in CRC in the hospital sewage system in 2023, and they reported the detection of 24.6% of *Citrobacter* strains carrying two carbapenemases when investigating hospital wastewater, in contrast to the detection of only 2% of strains carrying two carbapenemases in human-sourced strains (4). Based on these results and our findings on the high genetic diversity of isolates and the large number of isolates carrying one or more carbapenemases, we hypothesize that the marked increase in CRC in our hospital is a consequence of a self-perpetuating cycle: (i) CRC multiply in the sewage system; (ii) *Citrobacter* strains increasingly acquire carbapenem resistance through pronounced horizontal gene transfer; (iii) patients acquire CRC when using sanitary facilities; (iv) additionally, strains are sporadically passed on through patient-to-patient transmission; (v) there is a colonization of previously uncontaminated sewage pipes via patient excretions; and (vi) CRC multiply in the sewage system.

In our study, 65.1% of CRC patient strains were assigned to subclusters. The subcluster analysis demonstrated that in almost 75% of cases, the acquisition of isolates occurred without a documented epidemiological link between the patients concerned. These findings support the hypothesis that the environment constitutes an important reservoir for CRC acquisition. When wastewater samples from the bathrooms of three different wards were examined, CRC were detected in 23 out of 34 rooms (67.6%). The results revealed a heterogeneous pattern, consisting of many different strains that were genetically consistent with patient-derived strains (69.2%) as well as CRC strains not previously found in clinical patient samples. Transmission from the sewage system to the patient could be reconstructed in numerous cases. However, in many cases, the precise route of transmission could not be determined, likely due to transmission events occurring across multiple wards. These results are in some contrast to previous outbreak reports, in which only one strain localized in the sewage system of a single room or ward colonized or infected a large number of patients (10, 12). Our findings highlight a new, more complex situation, reflecting a highly diverse hospital microbiome that is spreading throughout many departments within our hospital.

The question arises as to which measures could be implemented to halt this development. Many methods for reducing the spread of highly resistant wastewater germs have been reported. These include cleaning and disinfection (34), biofilm disruption using vibration bundled with heat or ultraviolet radiation (35, 36), and replacement of sanitary facilities (34). At our hospital, the sanitary installations in the affected wards were renewed, and cleaning and disinfection procedures were intensified through the application of peracetic acid in the toilet and shower drains. Yet, none of the abovementioned approaches have demonstrated long-term efficacy. To develop innovative technologies that enhance patient safety, further research is paramount.

Our study has several limitations. Since this was a single-center study, the extent to which the results can be generalized is limited. Due to risk-based admission screening, not all patients were screened systematically. Furthermore, rectal swabs are known to have a reduced sensitivity. Environmental sampling was only conducted on selected wards. Further research should extend sampling to additional departments and wards. Sampling the sinks was not feasible due to technical difficulties. Given the substantial bacterial load in the wastewater, we were unable to identify all the colony-forming units present. Furthermore, bacterial populations can evolve over time, which may result in their inconsistent detectability in wastewater. Since the environmental investigation focused on the wastewater system, samples of surfaces and air were not collected. Finally, due to ethical regulations, we were unable to obtain additional patient information, such as infection symptoms or details of antibiotic treatment.

## Data Availability

The genome sequencing data are publicly available at NCBI GenBank under BioProject PRJNA1301355. Accession numbers are provided in File S7.

## SUPPLEMENTAL MATERIAL

File S1 spectrum.02670-25-s0001.pdf Best BLAST matches to PLSDB plasmids.

File S2 spectrum.02670-25-s0002.pdf Statistical analysis.

File S3 spectrum.02670-25-s0003.pdf Epidemiological links.

File S4 spectrum.02670-25-s0004.pdf Pairwise distance distributions.

File S5 spectrum.02670-25-s0005.pdf Room occupancy on ward X2.

File S6 spectrum.02670-25-s0006.pdf Patient room occupancy.

File S7 spectrum.02670-25-s0007.pdf Accession numbers.

ASM does not own the copyrights to Supplemental Material that may be linked to, or accessed through, an article. The authors have granted ASM a non-exclusive, world-wide license to publish the Supplemental Material files. Please contact the corresponding author directly for reuse.
