# Supplementary material for: Rising incidence of carbapenem-resistant Citrobacter spp. in a German tertiary-care hospital: epidemiology, clinical impact, and the role of the hospital wastewater system—findings from a six-year molecular study
Source: Microbiol Spectr. 2026 Jan 22;14(3):e02670-25. doi: 10.1128/spectrum.02670-25 (PMC12955474; doi:10.1128/spectrum.02670-25)
Supplement: File S1 — Best BLAST matches to PLSDB plasmids. [file spectrum.02670-25-s0001.pdf]

Supplemental File 1: Best BLAST matches to PLSDB plasmids

Isolate	Plasmid accession	Carbapenemase	Inc groups
CL18	NZ_CP018945.1	blaKPC-2	IncN
CL6	OW969779.1	blaKPC-2	IncFIB(K)_1_Kpn3 + IncX3 + IncFII_1_pKP91
KE10017	NZ_AP024759.1	blaKPC-2	ColRNAI
KE10036	NZ_CP026661.1	blaVIM-1	IncHI2A + IncHI2 + RepA_1_pKPC-CAV1321
KE10044	NC_011382.1	blaKPC-2	ColRNAI
KE10044	NZ_CP081819.1	blaNDM-5	IncX3
KE10055	NZ_AP022395.1	blaOXA-48	IncA/C2
KE10071	NZ_CP027051.1	blaKPC-2	IncX3
KE10091	NZ_AP024777.1	blaKPC-2	ColRNAI
KE10091	NZ_CP026661.1	blaVIM-1	IncHI2A + IncHI2 + RepA_1_pKPC-CAV1321
KE10099	CP025215.1	blaNDM-5	IncX3
KE10115	NZ_CP023897.1	blaOXA-181	IncX3 + ColKP3
KE10138	NZ_CP023897.1	blaOXA-181	IncX3 + ColKP3
KE10151	NZ_CP022150.1	blaOXA-48	IncL/M(pOXA-48)_1_pOXA-48
KE10167	NZ_AP024777.1	blaKPC-2	ColRNAI
KE10186	NZ_CP022150.1	blaOXA-48	IncL/M(pOXA-48)_1_pOXA-48
KE10204	NZ_CP022150.1	blaOXA-48	IncL/M(pOXA-48)_1_pOXA-48
KE10205	NZ_CP028565.1	blaGES-5	not detected
KE10217	NZ_CP023897.1	blaOXA-181	IncX3 + ColKP3
KE10233	NZ_CP023897.1	blaOXA-181	IncX3 + ColKP3
KE10263	NC_017097.1	blaKPC-2	IncQ2
KE10303	NZ_CP025009.1	blaKPC-2	IncFIB(K)_1_Kpn3 + ColRNAI + IncFII_1_pKP91
KE10388	NZ_CP022150.1	blaOXA-48	IncL/M(pOXA-48)_1_pOXA-48
KE10444	NC_011382.1	blaKPC-2	ColRNAI
KE10451	NC_011382.1	blaKPC-2	ColRNAI
KE10459	NZ_CP022351.1	blaNDM-5	IncX3
KE10459	NZ_CP022150.1	blaOXA-48	IncL/M(pOXA-48)_1_pOXA-48
KE10490	NZ_CP026241.1	blaKPC-2	IncFII(Yp)_1_Yersenia + IncFIB(pB171)_1_pB171
KE10504	NC_011382.1	blaKPC-2	ColRNAI
KE10581	NZ_AP024777.1	blaKPC-2	ColRNAI
KE10692	CP052359.1	blaKPC-2	IncN

KE10724	NZ_CP090630.1	blaKPC-3	IncFIB(pQil)_1_pQil + IncFII_1_pKP91
KE10742	CP052359.1	blaKPC-2	IncN
KE10748	NZ_CP022574.1	blaKPC-2	IncFII_1_pKP91 + IncFIB(K)_1_Kpn3
KE10748	NZ_CP022150.1	blaOXA-48	IncL/M(pOXA-48)_1_pOXA-48
KE10750	NC_020452.1	blaVIM-2	not detected
KE10750	NZ_CP022574.1	blaKPC-2	IncFII_1_pKP91 + IncFIB(K)_1_Kpn3
KE10755	NZ_CP022150.1	blaOXA-48	IncL/M(pOXA-48)_1_pOXA-48
KE10782	NZ_CP097343.1	blaKPC-2	IncHI2A + IncHI2 + RepA_1_pKPC-CAV1321
KE10793	NZ_AP024777.1	blaKPC-2	ColRNAI
KE10821	NZ_CP022150.1	blaOXA-48	IncL/M(pOXA-48)_1_pOXA-48
KE10867	CP025215.1	blaNDM-5	IncX3
KE10916	NZ_CP018945.1	blaKPC-2	IncN
KE10916	NZ_CP026661.1	blaVIM-1	IncHI2A + IncHI2 + RepA_1_pKPC-CAV1321
KE10937	CP052359.1	blaKPC-2	IncN
KE10941	CP025215.1	blaNDM-5	IncX3
KE10941	KP294350.1	blaKPC-2	not detected
KE10994	NZ_CP022351.1	blaNDM-5	IncX3
KE10994	NZ_CP022150.1	blaOXA-48	IncL/M(pOXA-48)_1_pOXA-48
KE10995	NC_017097.1	blaKPC-2	IncQ2
KE11015	NZ_CP022150.1	blaOXA-48	IncL/M(pOXA-48)_1_pOXA-48
KE11038	NZ_CP022574.1	blaKPC-2	IncFII_1_pKP91 + IncFIB(K)_1_Kpn3
KE11059	NZ_CP022150.1	blaOXA-48	IncL/M(pOXA-48)_1_pOXA-48
KE11060	NZ_CP097343.1	blaKPC-2	IncHI2A + IncHI2 + RepA_1_pKPC-CAV1321
KE11066	NC_011382.1	blaKPC-2	ColRNAI
KE11066	NZ_CP081819.1	blaNDM-5	IncX3
KE11079	NZ_CP018949.1	blaKPC-2	ColRNAI
KE11087	NZ_AP024777.1	blaKPC-2	ColRNAI
KE11116	NZ_CP055540.1	blaKPC-2	IncFII(S)
KE11116	NZ_CP022150.1	blaOXA-48	IncL/M(pOXA-48)_1_pOXA-48
KE11149	CP052359.1	blaKPC-2	IncN
KE11157	NZ_AP024777.1	blaKPC-2	ColRNAI
KE11166	NZ_KY271413.1	blaKPC-2	IncN
KE11166	NZ_CP026661.1	blaVIM-1	IncHI2A + IncHI2 + RepA_1_pKPC-CAV1321

KE11195	NZ_CP132285.1	blaKPC-2	not detected
KE11197	NZ_CP022150.1	blaOXA-48	IncL/M(pOXA-48)_1_pOXA-48
KE11218	NZ_AP024777.1	blaKPC-2	ColRNAI
KE11224	NZ_AP024777.1	blaKPC-2	ColRNAI
KE11227	NZ_CP056911.1	blaKPC-2	IncFII(S)
KE11242	NZ_CP022351.1	blaNDM-5	IncX3
KE11244	NZ_AP024759.1	blaKPC-2	ColRNAI
KE11254	NZ_CP022574.1	blaKPC-2	IncFII_1_pKP91 + IncFIB(K)_1_Kpn3
KE11280	NZ_AP024777.1	blaKPC-2	ColRNAI
KE11296	NZ_CP022351.1	blaNDM-5	IncX3
KE11297	NC_020452.1	blaVIM-2	not detected
KE11297	NZ_CP022574.1	blaKPC-2	IncFII_1_pKP91 + IncFIB(K)_1_Kpn3
KE11320	NC_017097.1	blaKPC-2	IncQ2
KE11321	CP025215.1	blaNDM-5	IncX3
KE11321	KP294350.1	blaKPC-2	not detected
KE11351	NZ_AP024777.1	blaKPC-2	ColRNAI
KE11368	NZ_AP024777.1	blaKPC-2	ColRNAI
KE11389	NZ_CP022574.1	blaKPC-2	IncFII_1_pKP91 + IncFIB(K)_1_Kpn3
KE11391	NZ_CP081819.1	blaNDM-5	IncX3
KE11391	NZ_CP022150.1	blaOXA-48	IncL/M(pOXA-48)_1_pOXA-48
KE11414	NZ_AP024759.1	blaKPC-2	ColRNAI
KE11415	OW969779.1	blaKPC-2	IncFIB(K)_1_Kpn3 + IncX3 + IncFII_1_pKP91
KE11455	NZ_AP024777.1	blaKPC-2	ColRNAI
KE11457	NZ_AP024777.1	blaKPC-2	ColRNAI
KE11474	KP294350.1	blaKPC-2	not detected
KE11474	NZ_CP096179.1	blaNDM-7	IncX3
KE11545	NZ_AP024759.1	blaKPC-2	ColRNAI
KE11546	NZ_CP055540.1	blaKPC-2	IncFII(S)
KE11553	NC_011382.1	blaKPC-2	ColRNAI
KE11553	NZ_CP022351.1	blaNDM-5	IncX3
KE11560	CP025215.1	blaNDM-5	IncX3
KE11568	NZ_CP026661.1	blaVIM-1	IncHI2A + IncHI2 + RepA_1_pKPC-CAV1321
KE11582	NC_011382.1	blaKPC-2	ColRNAI

KE11603	NZ_CP022150.1	blaOXA-48	IncL/M(pOXA-48)_1_pOXA-48
KE11609	OW969942.1	blaKPC-2	IncR
KE11620	NZ_CP077413.1	blaKPC-2	IncN
KE11648	NZ_CP081819.1	blaNDM-5	IncX3
KE11703	NZ_CP022150.1	blaOXA-48	IncL/M(pOXA-48)_1_pOXA-48
KE11719	NZ_AP024777.1	blaKPC-2	ColRNAI
KE11727	NZ_CP059691.1	blaKPC-2	IncN
KE11728	NZ_CP022574.1	blaKPC-2	IncFII_1_pKP91 + IncFIB(K)_1_Kpn3
KE11759	NZ_CP022351.1	blaNDM-5	IncX3
KE11761	OW969942.1	blaKPC-2	IncR
KE11771	NZ_AP024777.1	blaKPC-2	ColRNAI
KE11774	NZ_CP028577.1	blaNDM-5	IncX3
KE11774	CP085739.1	blaVIM-1	IncHI1A(CIT)_1_pNDM-CIT + IncHI1B(CIT)_1_pNDM-CIT
KE11774	NZ_CP022150.1	blaOXA-48	IncL/M(pOXA-48)_1_pOXA-48
KE11782	NZ_CP056186.1	blaKPC-2	IncFII(S)
KE11789	NZ_CP022150.1	blaOXA-48	IncL/M(pOXA-48)_1_pOXA-48
KE11799	NZ_CP077413.1	blaKPC-2	IncN
KE11807	NZ_CP022150.1	blaOXA-48	IncL/M(pOXA-48)_1_pOXA-48
KE11835	KP294350.1	blaKPC-2	not detected
KE11837	NZ_CP056186.1	blaKPC-2	IncFII(S)
KE11841	NZ_CP022150.1	blaOXA-48	IncL/M(pOXA-48)_1_pOXA-48
KE11856	NZ_AP024777.1	blaKPC-2	ColRNAI
KE11863	OW969942.1	blaKPC-2	IncR
KE11898	NZ_AP024777.1	blaKPC-2	ColRNAI
KE11904	NZ_CP022150.1	blaOXA-48	IncL/M(pOXA-48)_1_pOXA-48
KE11922	NZ_CP018945.1	blaKPC-2	IncN
KE11940	NZ_CP022351.1	blaNDM-5	IncX3
KE9314	NZ_CP022351.1	blaNDM-5	IncX3
KE9378	NZ_AP024777.1	blaKPC-2	ColRNAI
KE9511	NZ_AP024777.1	blaKPC-2	ColRNAI
KE9511	NZ_CP026661.1	blaVIM-1	IncHI2A + IncHI2 + RepA_1_pKPC-CAV1321
KE9554	NZ_CP022150.1	blaOXA-48	IncL/M(pOXA-48)_1_pOXA-48
KE9559	NZ_CP026661.1	blaVIM-1	IncHI2A + IncHI2 + RepA_1_pKPC-CAV1321

KE9650	NZ_CP022351.1	blaNDM-5	IncX3
KE9724	NZ_CP022150.1	blaOXA-48	IncL/M(pOXA-48)_1_pOXA-48
KE9724	NZ_CP009115.1	blaNDM-1	IncFII_1_pKP91 + IncFIB(pQil)_1_pQil
KE9752	NZ_CP026661.1	blaVIM-1	IncHI2A + IncHI2 + RepA_1_pKPC-CAV1321
KE9775	NZ_AP024777.1	blaKPC-2	ColRNAI
KE9784	NZ_CP081819.1	blaNDM-5	IncX3
KE9790	NZ_CP081819.1	blaNDM-5	IncX3
KE9831	NZ_CP023897.1	blaOXA-181	IncX3 + ColKP3
KE9890	NZ_OQ803524.1	blaKPC-2	IncFII(S)
KE9957	NZ_CP081819.1	blaNDM-5	IncX3
KE9961	KP294350.1	blaKPC-2	not detected
KE9999	NZ_AP024777.1	blaKPC-2	ColRNAI
KE9999	NZ_CP026661.1	blaVIM-1	IncHI2A + IncHI2 + RepA_1_pKPC-CAV1321
KT1073	NZ_CP022351.1	blaNDM-5	IncX3
KT1094	NZ_CP022351.1	blaNDM-5	IncX3
KT1094	NZ_CP022150.1	blaOXA-48	IncL/M(pOXA-48)_1_pOXA-48
KT1099	NC_011382.1	blaKPC-2	ColRNAI
KT1099	NZ_CP081819.1	blaNDM-5	IncX3
KT1100	KP294350.1	blaKPC-2	not detected
KT1100	NZ_CP022351.1	blaNDM-5	IncX3
KT1107	CP025215.1	blaNDM-5	IncX3
KT1108	NZ_CP028577.1	blaNDM-5	IncX3
KT1315	NZ_AP024777.1	blaKPC-2	ColRNAI
KT1317	NZ_AP024777.1	blaKPC-2	ColRNAI
KT1318	NZ_AP024777.1	blaKPC-2	ColRNAI
KT1319	NC_017097.1	blaKPC-2	IncQ2
KT1320	NC_017097.1	blaKPC-2	IncQ2
KT1323	NZ_AP024777.1	blaKPC-2	ColRNAI
KT1324	NZ_AP024777.1	blaKPC-2	ColRNAI
KT1325	NZ_AP024777.1	blaKPC-2	ColRNAI
KT1326	NZ_AP024777.1	blaKPC-2	ColRNAI
KT1326	NZ_CP081819.1	blaNDM-5	IncX3
KT1344	NZ_AP024777.1	blaKPC-2	ColRNAI

KT1346	NC_011382.1	blaKPC-2	ColRNAI
KT1347	NC_017097.1	blaKPC-2	IncQ2
KT1348	NC_017097.1	blaKPC-2	IncQ2
KT1358	NZ_AP024777.1	blaKPC-2	ColRNAI
KT1359	NZ_AP024777.1	blaKPC-2	ColRNAI
KT1360	NZ_AP024777.1	blaKPC-2	ColRNAI
KT1367	NC_011382.1	blaKPC-2	ColRNAI
KT1441	NZ_CP018945.1	blaKPC-2	IncN
KT1443	NC_017097.1	blaKPC-2	IncQ2
KT1444	NZ_AP024777.1	blaKPC-2	ColRNAI
KT1445	CP052359.1	blaKPC-2	IncN
KT1446	KP294350.1	blaKPC-2	not detected
KT1621	NZ_CP022351.1	blaNDM-5	IncX3
KT1622	NC_011382.1	blaKPC-2	ColRNAI
KT1622	NZ_CP022351.1	blaNDM-5	IncX3
KT1622	NZ_CP022150.1	blaOXA-48	IncL/M(pOXA-48)_1_pOXA-48
KT1623	NZ_CP022574.1	blaKPC-2	IncFII_1_pKP91 + IncFIB(K)_1_Kpn3
KT1624	NZ_AP024777.1	blaKPC-2	ColRNAI
KT1625	NZ_KY659387.1	blaKPC-2	IncX3
KT1627	NZ_CP018945.1	blaKPC-2	IncN
