# Supplementary material for: Rising incidence of carbapenem-resistant Citrobacter spp. in a German tertiary-care hospital: epidemiology, clinical impact, and the role of the hospital wastewater system—findings from a six-year molecular study
Source: Microbiol Spectr. 2026 Jan 22;14(3):e02670-25. doi: 10.1128/spectrum.02670-25 (PMC12955474; doi:10.1128/spectrum.02670-25)
Supplement: File S2 — Statistical analysis. [file spectrum.02670-25-s0002.pdf]

Supplemental File 2: Statistical analysis

Section A

Table A1: Raw yearly counts of carbapenem-resistant *Enterobacterales* (CRE) for figures 1a) and b). For *Citrobacter* spp. the number of screening (S) and diagnostic (D) specimens is given in parentheses as (S/D)

Year	Patient-Days	CRE	Number of isolates
2019	508360	*Citrobacter* spp.	7 (5/2)
		Enterobacter cloacae complex	35
		Escherichia coli	15
		Klebsiella pneumoniae	19
2020	482920	*Citrobacter* spp.	21 (11/10)
		Enterobacter cloacae complex	32
		Escherichia coli	12
		Klebsiella pneumoniae	21
2021	489356	*Citrobacter* spp.	18 (12/6)
		Enterobacter cloacae complex	18
		Escherichia coli	14
		Klebsiella pneumoniae	24
2022	485201	*Citrobacter* spp.	17 (14/3)
		Enterobacter cloacae complex	21
		Escherichia coli	14
		Klebsiella pneumoniae	19
2023	501870	*Citrobacter* spp.	33 (27/6)
		Enterobacter cloacae complex	19
		Escherichia coli	17
		Klebsiella pneumoniae	23
2024	470354	*Citrobacter* spp.	42 (31/11)
		Enterobacter cloacae complex	23
		Escherichia coli	17
		Klebsiella pneumoniae	29

Poisson regression model with patient-days as offset and CRE, time since 2019 and their interaction as covariates, i.e.

$$\text{Number of isolates} = \log(\text{Patient-Days}) + \beta_0 + \beta_1 \text{Time since 2019} + \beta_2 \text{CRE} + \beta_3 \text{Time since 2019} \cdot \text{CRE}.$$

Table A2: Results of the Poisson model for the incidence rate per patient-day. For carbapenem-resistant *Enterobacterales* the reference category is *Citrobacter* spp.

	Estimate	SE	Statistic	p-value
(Intercept)	-10.776	0.185	-58.394	0.000
Enterobacter cloacae complex	1.121	0.224	4.998	0.000
Escherichia coli	0.227	0.262	0.866	0.399
Klebsiella pneumoniae	0.599	0.240	2.497	0.024
time	0.281	0.051	5.533	0.000
Enterobacter cloacae complex:time	-0.385	0.069	-5.602	0.000
Escherichia coli:time	-0.225	0.078	-2.884	0.011
Klebsiella pneumoniae:time	-0.208	0.070	-2.975	0.009

Table A3: Model based time trend for the incidence rate per patient-day of the different carbapenem-resistant *Enterobacterales* (CRE)

CRE	Time trend	Lower limit CI	Upper limit CI
Citrobacter spp.	1.325	1.199	1.464
Enterobacter cloacae complex	0.901	0.823	0.987
Escherichia coli	1.058	0.942	1.188
Klebsiella pneumoniae	1.076	0.979	1.182

Table A4: Observed incidence rate per 100,000 patient-days. Model based predicted rates are given in parantheses.

CRE	2019	2020	2021	2022	2023	2024
Citrobacter spp.	1.38 (2.09)	4.35 (2.77)	3.68 (3.67)	3.5 (4.86)	6.58 (6.44)	8.93 (8.53)
Enterobacter cloacae complex	6.88 (6.41)	6.63 (5.78)	3.68 (5.21)	4.33 (4.7)	3.79 (4.23)	4.89 (3.82)
Escherichia coli	2.95 (2.62)	2.48 (2.78)	2.86 (2.94)	2.89 (3.11)	3.39 (3.28)	3.61 (3.47)
Klebsiella pneumoniae	3.74 (3.81)	4.35 (4.09)	4.9 (4.4)	3.92 (4.74)	4.58 (5.1)	6.17 (5.48)

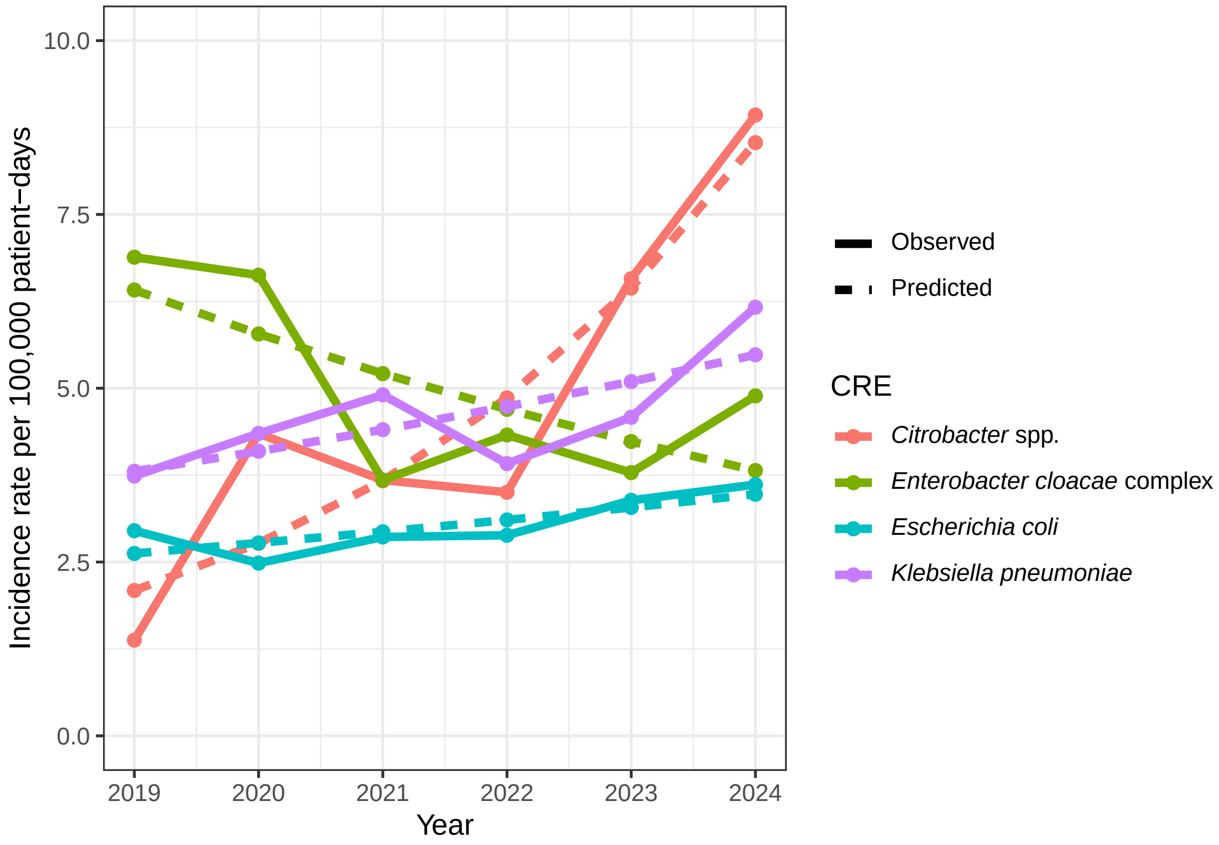

Figure A1: Annual incidence rates of carbapenem-resistant *Citrobacter* spp., *Enterobacter cloacae* complex, *Escherichia coli* and *Klebsiella pneumoniae* from 2019 to 2024

Section B

Poisson regression model with patient-days as offset and CRE, time since 2019 and their interaction as covariates adjusted for the outbreak of *Enterobacter cloacae* complex, i.e.

Number of isolates = log(Patient-Days)+β_0+β_1Time since 2019+β_2CRE+β_3Time since 2019·CRE+β_4Outbreak.

Table B1: Results of the Poisson model for the incidence rate per patient-day, adjusted for the *Enterobacter cloacae* complex outbreak. For carbapenem-resistant *Enterobacterales* the reference category is *Citrobacter* spp.

	Estimate	SE	Statistic	p-value
(Intercept)	-10.776	0.162	-66.709	0.000
Enterobacter cloacae complex	0.486	0.332	1.465	0.164
Escherichia coli	0.227	0.229	0.990	0.338
Klebsiella pneumoniae	0.599	0.210	2.853	0.012
time	0.281	0.045	6.321	0.000
outbreak	0.659	0.273	2.412	0.029
Enterobacter cloacae complex:time	-0.224	0.089	-2.514	0.024
Escherichia coli:time	-0.225	0.068	-3.294	0.005
Klebsiella pneumoniae:time	-0.208	0.061	-3.398	0.004

Table B2: Model based time trend for the incidence rate per patient-day of the different carbapenem-resistant *Enterobacterales* (CRE) if no outbreak had happened.

CRE	Time trend	Lower limit CI	Upper limit CI
Citrobacter spp.	1.325	1.214	1.446
Enterobacter cloacae complex	1.059	0.910	1.232
Escherichia coli	1.058	0.956	1.171
Klebsiella pneumoniae	1.076	0.990	1.168

Table B3: Observed incidence rate per 100,000 patient-days. Model based predicted rates if no outbreak had occured (NO) and if an outbreak had occurred (O) are given in parantheses as (NO/O).

CRE	2019	2020	2021	2022	2023	2024
Citrobacter spp.	1.38 (2.09/4.04)	4.35 (2.77/5.35)	3.68 (3.67/7.09)	3.5 (4.86/9.4)	6.58 (6.44/12.45)	8.93 (8.53/16.49)
Enterobacter cloacae complex	6.88 (3.4/6.57)	6.63 (3.6/6.96)	3.68 (3.81/7.37)	4.33 (4.04/7.8)	3.79 (4.28/8.26)	4.89 (4.53/8.75)
Escherichia coli	2.95 (2.62/5.07)	2.48 (2.78/5.36)	2.86 (2.94/5.67)	2.89 (3.11/6)	3.39 (3.28/6.35)	3.61 (3.47/6.72)
Klebsiella pneumoniae	3.74 (3.81/7.36)	4.35 (4.09/7.91)	4.9 (4.4/8.51)	3.92 (4.74/9.16)	4.58 (5.1/9.85)	6.17 (5.48/10.59)

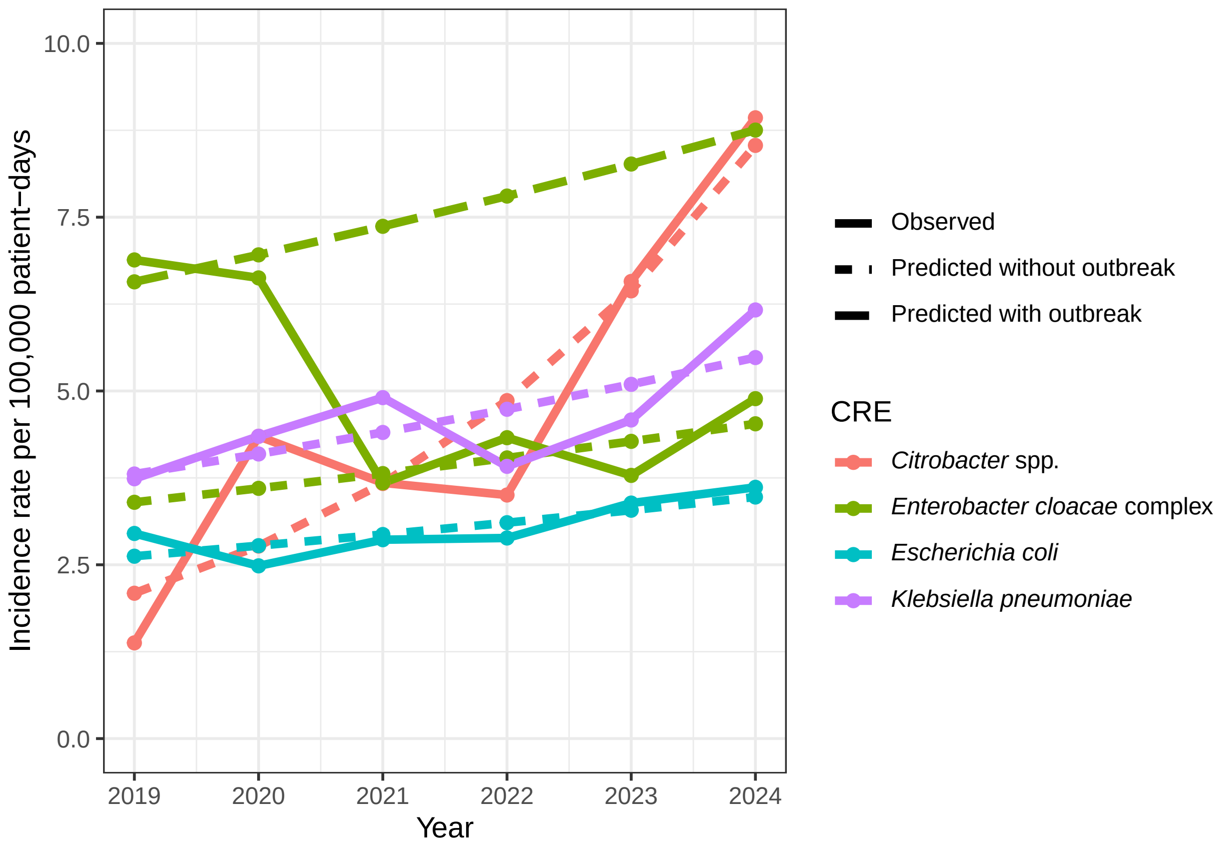

Figure B1: Annual incidence rates of carbapenem-resistant *Citrobacter* spp., *Enterobacter cloacae* complex, *Escherichia coli* and *Klebsiella pneumoniae* from 2019 to 2024, adjusted for the *Enterobacter cloacae* complex outbreak

Section C

The number of screening tests performed within each calendar year under investigation is shown in the following table.

Table C1: Number of screening tests performed within each calendar year

Year	Screening tests
2019	26093
2020	25928
2021	26101
2022	25509
2023	25875
2024	25497

Poisson regression model with patient-days and number of screening tests as offset and CRE, time since 2019 and their interaction as covariates adjusted for the outbreak of *Enterobacter cloacae* complex, i.e.

$$\text{Number of isolates} = \log(\text{Patient-Days} \cdot \text{Screening-tests}) + \beta_0 + \beta_1 \text{Time since 2019} + \beta_2 \text{CRE} + \beta_3 \text{Time since 2019} \cdot \text{CRE} + \beta_4 \text{Outbreak}.$$

Table C2: Results of the Poisson model for the incidence rate per patient-day and screeningtest, adjusted for the *Enterobacter cloacae* complex outbreak. For carbapenem-resistant *Enterobacterales* the reference category is *Citrobacter* spp.

	Estimate	SE	Statistic	p-value
(Intercept)	-20.946	0.161	-129.962	0.000
Enterobacter cloacae complex	0.480	0.331	1.452	0.167
Escherichia coli	0.227	0.229	0.994	0.336
Klebsiella pneumoniae	0.599	0.210	2.861	0.012
time	0.286	0.044	6.430	0.000
outbreak	0.666	0.272	2.444	0.027
Enterobacter cloacae complex:time	-0.223	0.089	-2.506	0.024
Escherichia coli:time	-0.225	0.068	-3.303	0.005
Klebsiella pneumoniae:time	-0.208	0.061	-3.408	0.004

Table C3: Model based time trend of the incidence rate per patient-day and screeningtest of the different carbapenem-resistant *Enterobacterales* (CRE) if no outbreak had happened.

CRE	Time trend	Lower limit CI	Upper limit CI
Citrobacter spp.	1.330	1.220	1.451
Enterobacter cloacae complex	1.065	0.916	1.238
Escherichia coli	1.062	0.960	1.176
Klebsiella pneumoniae	1.080	0.995	1.173

Table C4: Observed incidence rate per 10,000 screeningtests and 100,000 patient-days. Model based predicted rates if no outbreak had occured (NO) and if an outbreak had occurred (O) are given in parantheses as (NO/O).

CRE	2019	2020	2021	2022	2023	2024
Citrobacter spp.	0.53 (0.8/1.56)	1.68 (1.07/2.07)	1.41 (1.42/2.76)	1.37 (1.89/3.67)	2.54 (2.51/4.88)	3.5 (3.34/6.49)
Enterobacter cloacae complex	2.64 (1.29/2.52)	2.56 (1.38/2.68)	1.41 (1.47/2.86)	1.7 (1.56/3.04)	1.46 (1.67/3.24)	1.92 (1.77/3.45)
Escherichia coli	1.13 (1.01/1.96)	0.96 (1.07/2.08)	1.1 (1.13/2.21)	1.13 (1.2/2.34)	1.31 (1.28/2.49)	1.42 (1.36/2.64)
Klebsiella pneumoniae	1.43 (1.46/2.84)	1.68 (1.57/3.06)	1.88 (1.7/3.31)	1.54 (1.84/3.58)	1.77 (1.98/3.86)	2.42 (2.14/4.17)

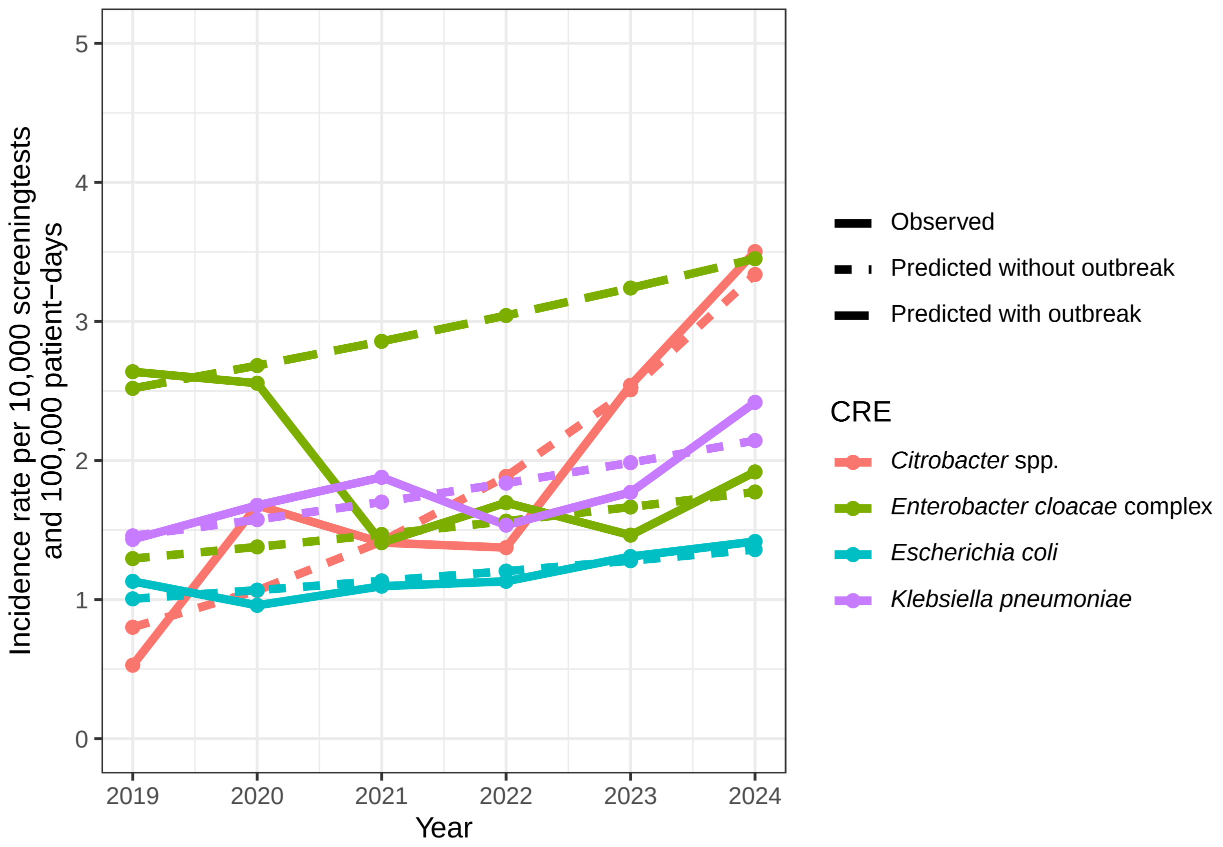

Figure C1: Annual incidence rates of carbapenem-resistant *Citrobacter* spp., *Enterobacter cloacae* complex, *Escherichia coli* and *Klebsiella pneumoniae* from 2019 to 2024, adjusted for the yearly counts of screeningtests and the *Enterobacter cloacae* complex outbreak
