# Supplementary material for: Rising incidence of carbapenem-resistant Citrobacter spp. in a German tertiary-care hospital: epidemiology, clinical impact, and the role of the hospital wastewater system—findings from a six-year molecular study
Source: Microbiol Spectr. 2026 Jan 22;14(3):e02670-25. doi: 10.1128/spectrum.02670-25 (PMC12955474; doi:10.1128/spectrum.02670-25)
Supplement: File S3 — Epidemiological links. [file spectrum.02670-25-s0003.pdf]

Supplemental File 3: Epidemiological links

Subcluster	Patient	Date of Admission	Date of Discharge	Overlapping Hospital Stay*	Clinic	Department	Ward
A	1A	02/19/20	04/21/20	No	-	-	-
	2A	01/28/21	02/18/21	No	-	-	-
	3A	04/28/22<br>06/02/22<br>07/14/22<br>10/10/22<br>11/09/22<br>11/23/22<br>12/14/22<br>01/03/23<br>02/13/23<br>03/03/23<br>05/03/23<br>06/07/23<br>06/30/23<br>07/24/23<br>08/15/23<br>09/08/23<br>09/29/23<br>10/20/23<br>12/22/23<br>02/01/24<br>03/28/24<br>06/21/24<br>09/20/24<br>12/13/24	05/10/22<br>06/28/22<br>09/30/22<br>11/03/22<br>11/10/22<br>11/28/22<br>12/21/22<br>02/06/23<br>02/21/23<br>04/28/23<br>05/31/23<br>06/13/23<br>07/09/23<br>08/01/23<br>08/22/23<br>09/13/23<br>10/09/23<br>12/15/23<br>12/25/23<br>03/01/24<br>03/30/24<br>06/24/24<br>09/21/24<br>12/14/24	Yes, with patient 4 (03/24/23 - 03/31/23)	Internal Medicine	-	-
				Yes, with patient 5 (11/23/22 - 11/28/22)	Internal Medicine		
				Yes, with patient 6 (09/20/24 - 09/21/24)	Internal Medicine	D1	I7
				Yes, with patient 7 (10/24/23 - 12/05/23)	Internal Medicine	-	-
				Yes, with patient 8 (11/19/23 - 12/05/23)	Internal Medicine		
				Yes, with patient 9 (02/02/24 - 02/19/24)	Internal Medicine		
				Yes, with patient 10 (02/07/24 - 02/13/24)	Internal Medicine	D1	
				Yes, with patient 11 (08/15/23 - 08/22/23)	Internal Medicine	D1	I7
	4A	03/24/23<br>04/24/23	03/31/23<br>05/02/23	Yes, with patient 3 (03/24/23 - 03/31/23)	Surgery	-	-

Supplemental File 3: Epidemiological links

		06/12/23<br>07/31/23	06/28/23<br>08/10/23	Yes, with patient 5 (06/20/23 - 06/28/23)	Surgery	D10	-
				Yes, with patient 12 (06/23/23 - 06/28/23)	Surgery	-	-
	5A	11/10/22<br>12/01/22<br>06/20/23<br>02/18/24	11/28/22<br>12/07/22<br>08/07/23<br>02/23/24	Yes, with patient 3 (11/23/22 - 11/28/22)	Surgery	-	-
				Yes, with patient 4 (06/20/23 - 06/28/23)	Surgery	D8	-
				Yes, with patient 9 (02/18/24 -02/19/24)	Neurology	-	-
				Yes, with patient 12 (06/23/23 - 06/28/23)	Surgery		
	6A	09/16/23	09/21/23	Yes, with patient 3 (09/20/24 - 09/21/24)	Internal Medicine	D1	I7
				Yes, with patient 11 (09/19/23 - 09/20/23)	Internal Medicine	D1	I7
	7A	10/24/23<br>04/27/24	12/05/23<br>06/28/24	Yes, with patient 3 (10/24/23 - 12/05/23)	Surgery	-	-
				Yes, with patient 8 (11/19/23 - 12/05/23)	Surgery	D9	-
				Yes, with patient 9 (05/13/24 - 05/19/24)	Surgery	D8	-
				Yes, with patient 11 (06/19/24 - 06/28/24)	Surgery	D8	
	8A	11/19/23	12/05/23	Yes, with patient 3 (11/19/23 - 12/05/23)	Surgery	-	-
				Yes, with patient 7 (11/19/23 - 12/05/23)	Surgery	D8	-
	9A	01/11/24<br>05/13/24	02/19/24<br>05/19/24	Yes, with patient 3 (02/02/24 - 02/19/24)	Surgery	-	-

Supplemental File 3: Epidemiological links

		05/30/25	06/01/25	Yes, with patient 5 (02/18/24 - 02/19/24)	Surgery	-	-
				Yes, with patient 7 (05/13/24 - 05/19/24)	Surgery	D9	-
				Yes, with patient 10 (01/12/24 - 01/23/24)	Surgery	-	-
	10A	12/27/23<br>01/12/24<br>01/25/24<br>02/07/24<br>02/14/24	12/31/23<br>01/23/24<br>01/29/24<br>02/13/24<br>02/16/24	Yes, with patient 3 (02/07/24 - 02/13/24)	Internal Medicine	D3	-
				Yes, with patient 9 (01/12/24 - 01/23/24)	Internal Medicine	-	-
	11A	08/14/23<br>09/19/23<br>06/05/24<br>08/02/24	08/23/23<br>09/20/23<br>07/08/24<br>08/24/24	Yes, with patient 3 (08/15/23 - 08/22/23)	Internal Medicine	D1	I7
				Yes, with patient 6 (09/19/23 - 09/20/23)	Internal Medicine	D1	I7
				Yes, with patient 7 (06/19/24 - 06/28/24)	Surgery	D10	-
				Yes, with patient 12 (08/12/24 - 08/24/24)	Internal Medicine	D1	-
	12A	06/23/23<br>08/11/24	06/28/23<br>09/02/24	Yes, with patient 4 from 06/23/23 - 06/28/23	Internal Medicine	-	-
				Yes, with patient 5 from 06/23/23 - 06/28/23	Internal Medicine	-	-
				Yes, with patient 11 (08/12/24 - 08/24/24)	Internal Medicine	D2	-
	13A	11/17/24<br>12/02/24	11/20/24<br>12/10/24	No	-	-	-

Supplemental File 3: Epidemiological links

**B**	1B	08/23/20	11/16/20	No	-	-	-
	2B	01/10/20<br>07/29/23<br>08/09/23<br>08/16/23<br>03/27/24	02/07/20<br>08/02/23<br>08/11/23<br>08/31/23<br>03/28/24	Yes, with patient 4 (03/27/24 to 03/28/24)	Internal Medicine	D2	IMC12
	3B	12/25/23<br>01/12/24	01/08/24<br>01/22/24	No	-	-	-
	4B	03/06/24	05/10/24	Yes, with patient 2 (03/27/24 - 03/28/24)	Internal Medicine	D2	IMC12
	5B	08/02/24	08/27/24	No	-	-	-
	6B	09/25/24	10/23/24	No	-	-	-
**C**	1C	10/04/20	10/07/20	No	-	-	-
	2C	06/01/21<br>06/17/21	06/07/21<br>07/01/21	Yes, with patient 3 (06/01/21 - 06/28/21)	Internal Medicine	D5	-
	3C	03/10/21<br>03/27/21<br>04/09/22<br>05/30/22<br>12/08/22<br>03/16/23<br>08/25/23<br>10/10/23<br>12/19/23<br>03/05/24	03/15/21<br>08/26/21<br>04/23/22<br>06/17/22<br>12/09/22<br>03/23/23<br>09/08/23<br>10/13/23<br>12/24/23<br>03/19/24	Yes, with patient 2 (06/01/21 - 06/28/21)	Internal Medicine	D1	-
				Yes, with patient 4 (04/13/21 - 04/19/21, 03/16/21 – 03/21/23)	Internal Medicine	D6	-
				Yes, with patient 5 (08/20/21 – 08/26/21, 04/14/22 to 04/23/22, 06/05/22 – 06/08/22)	Internal Medicine	D6	I4
	4C	04/09/20<br>12/01/20<br>01/11/21	04/14/20<br>12/08/20<br>01/14/21	Yes, with patient 3 (04/13/21 - 04/19/21, 03/16/21 – 03/21/23)	Internal Medicine	D1	-

Supplemental File 3: Epidemiological links

		04/13/21<br>09/10/21<br>10/14/21<br>02/15/22<br>09/16/22<br>03/14/23<br>07/28/23<br>08/17/23<br>11/22/23	04/19/21<br>09/20/21<br>10/18/21<br>02/23/22<br>09/21/22<br>03/21/23<br>08/01/23<br>08/18/23<br>11/23/23	Yes, with patient 5 (09/10/21 – 09/20/21, 10/14/21 – 10/18/21)	Internal Medicine	D1	I7
	5C	08/20/21<br>03/03/22<br>04/14/22<br>05/11/22<br>06/05/22<br>11/14/22	01/20/22<br>03/16/22<br>04/26/22<br>05/13/22<br>06/08/22<br>11/16/22	Yes, with patient 3 (08/20/21 – 08/26/21, 04/14/22 to 04/23/22, 06/05/22 – 06/08/22)	(Surgery, Orthopedics), Internal Medicine	D6	I5
				Yes, with patient 4 (09/10/21 – 09/20/21, 10/14/21 – 10/18/21)	Internal Medicine	D1	I7
	6C	12/02/21	12/22/21	No	-	-	-
**D**	1D	03/17/20<br>10/09/20<br>10/23/20<br>11/23/20<br>05/10/21<br>06/14/21<br>10/14/21<br>05/13/22	03/24/20<br>10/14/20<br>10/27/20<br>11/24/20<br>05/11/21<br>06/21/21<br>10/25/21<br>05/17/22	Yes, with patient 2 (03/17/20 – 03/24/20)	Internal Medicine	-	-
	2D	02/13/20	04/16/20	Yes, with patient 1 (03/17/20 – 03/24/20)	Urology	-	-
	3D	08/10/20	08/25/20	No	-	-	-
	4D	04/22/20<br>01/24/21	04/29/20<br>02/03/21	Yes, with patient 5 (09/23/24 - 10/09/24)	Surgery	-	-

Supplemental File 3: Epidemiological links

		02/07/21<br>03/10/21<br>05/03/21<br>05/31/21<br>06/28/21<br>11/19/21<br>08/28/24	02/15/21<br>03/11/21<br>05/04/21<br>06/01/21<br>06/29/21<br>11/20/21<br>10/12/24				
	5D	09/23/24	10/09/24	Yes, with patient 4 (09/23/24 - 10/09/24)	Internal Medicine	-	-
**E**	1E	04/05/21<br>04/12/21<br>04/19/21<br>05/03/21<br>05/10/21<br>05/17/21<br>05/25/21<br>06/08/21<br>06/23/21<br>06/30/21<br>07/19/21<br>09/08/21<br>09/29/21<br>10/20/21	04/07/21<br>04/13/21<br>04/20/21<br>05/04/21<br>05/12/21<br>05/18/21<br>06/03/21<br>06/09/21<br>06/28/21<br>07/02/21<br>07/28/21<br>09/12/21<br>10/03/21<br>10/24/21	Yes, with patient 2 (09/29/21 – 10/03/21)	Internal Medicine	-	-
	2E	09/29/21<br>11/10/21<br>11/24/21<br>12/06/21<br>12/21/21<br>01/13/22<br>01/31/22<br>02/15/22<br>03/01/22<br>08/29/22<br>08/03/23	10/18/21<br>11/12/21<br>11/30/21<br>12/13/21<br>12/28/21<br>01/18/22<br>02/04/22<br>02/18/22<br>03/04/22<br>09/01/22<br>08/04/23	Yes, with patient 1 (09/29/21 – 10/03/21)	Surgery	-	-

Supplemental File 3: Epidemiological links

	3E	11/08/22<br>01/10/23<br>01/23/23<br>02/16/23<br>06/27/23<br>08/06/23<br>12/17/23<br>01/25/24<br>03/19/24<br>04/02/24<br>07/15/24<br>10/16/24<br>12/03/24	01/08/23<br>01/20/23<br>01/31/23<br>03/17/23<br>07/11/23<br>08/10/23<br>12/20/23<br>02/05/24<br>03/21/24<br>04/24/24<br>07/29/24<br>11/18/24<br>12/20/24	Yes, with patient 4 (03/09/23 - 03/11/23)	Internal Medicine	D3	O1
	4E	03/09/23	03/29/23	Yes, with patient 3 (03/09/23 - 03/11/23)	Internal Medicine	D3	O1
	5E	10/27/23	11/15/23	No	-	-	-
**F**	1F	01/08/20<br>01/31/20<br>03/09/20<br>04/08/20<br>04/28/20<br>05/10/20<br>04/24/20<br>07/22/20<br>09/01/20<br>09/13/20<br>10/23/20<br>01/07/21<br>02/16/21<br>07/28/21<br>08/29/21<br>10/06/21	01/15/20<br>02/07/20<br>03/17/20<br>04/18/20<br>05/08/20<br>05/17/20<br>06/07/20<br>07/30/20<br>09/04/20<br>09/22/20<br>10/30/20<br>01/14/21<br>03/04/21<br>08/05/21<br>10/01/21<br>10/10/21	Yes, with patient 4 (02/16/21 – 03/04/21)	Internal Medicine	D5	-

Supplemental File 3: Epidemiological links

	2F	12/30/20	01/25/21	Yes, with patient 3 (12/30/20 – 01/25/21)	Internal Medicine	D1	I1
	3F	12/15/20	01/25/21	Yes, with patient 2 (12/30/20 – 01/25/21)	Internal Medicine	D1	I4
	4F	02/03/21	04/20/21	Yes, with patient 1 (02/16/21 – 03/04/21)	Internal Medicine	D1	-
				Yes, with patient 5 (03/26/21 – 04/20/21)	Hemato Oncology	-	H3
	5F	03/26/21<br>05/19/21	05/05/21<br>05/22/21	Yes, with patient 4 (03/26/21 – 04/20/21)	Hemato Oncology	-	H1
**G**	1G	02/25/20<br>06/10/20<br>06/26/20<br>03/31/21<br>05/12/21<br>07/12/21	03/10/20<br>06/23/20<br>12/02/20<br>04/06/21<br>05/13/21<br>07/13/21	No	-	-	-
	2G	02/15/21<br>12/12/22	02/17/21<br>12/25/22	No	-	-	-
	3G	02/18/23<br>07/12/23<br>09/04/23<br>10/09/23<br>08/01/24<br>08/30/24<br>09/16/24<br>11/22/24	03/22/23<br>08/02/23<br>09/09/23<br>11/21/23<br>08/02/24<br>08/31/24<br>09/22/24<br>11/25/24	No	-	-	-
	4G	02/04/24<br>04/08/24<br>05/06/24<br>05/21/24<br>05/28/24<br>06/11/24	04/05/24<br>04/15/24<br>05/17/24<br>05/23/24<br>06/05/24<br>06/19/24	No	-	-	-

Supplemental File 3: Epidemiological links

**H**	1H	03/19/19	05/29/19	No	-		
	2H	08/05/19	10/08/19	No	-		
	3H	11/06/22	11/30/22	Yes, with patient 4 (11/06/22 – 11/29/22)	Urology		
	4H	09/21/22<br>04/17/23<br>05/22/23<br>06/21/23	11/29/22<br>05/04/23<br>06/13/23<br>06/23/23	Yes, with patient 3 (11/06/22 – 11/29/22)	Internal Medicine		

*All inpatient and outpatient stays since 2019 were reviewed, outpatient visits were not listed separately, as there were no concurrent stays. Concordant, overlapping patient stays were highlighted in the same color.
