# Supplementary material for: Rising incidence of carbapenem-resistant Citrobacter spp. in a German tertiary-care hospital: epidemiology, clinical impact, and the role of the hospital wastewater system—findings from a six-year molecular study
Source: Microbiol Spectr. 2026 Jan 22;14(3):e02670-25. doi: 10.1128/spectrum.02670-25 (PMC12955474; doi:10.1128/spectrum.02670-25)
Supplement: File S4 — Pairwise distance distributions. [file spectrum.02670-25-s0004.pdf]

Supplemental File 4: Pairwise distance distributions from patient isolates (KE) to environmental isolates (KT or CL)

| from | to | SNPs | VLKC | MLST | Subcluster |
|---|---|---|---|---|---|
| KE10044 | KT1069 | 10 | 1 | 22 | 1.2 |
| KE10044 | KT1099 | 2 | 1 | 22 | 1.2 |
| KE11066 | KT1069 | 13 | 1 | 22 | 1.2 |
| KE11066 | KT1099 | 7 | 1 | 22 | 1.2 |
| KE11689 | KT1069 | 13 | 1 | 22 | 1.2 |
| KE11689 | KT1099 | 13 | 1 | 22 | 1.2 |
| KE11296 | KT1094 | 2 | 1 | 22 | 1.8 |
| KE11296 | KT1622 | 3 | 1 | 22 | 1.8 |
| KE11391 | KT1094 | 1 | 1 | 22 | 1.8 |
| KE11391 | KT1622 | 2 | 1 | 22 | 1.8 |
| KE11759 | KT1094 | 2 | 1 | 22 | 1.8 |
| KE11759 | KT1622 | 5 | 1 | 22 | 1.8 |
| KE10036 | CL6 | 18 | 10 | 908 | 10.1 |
| KE11415 | CL6 | 2 | 10 | 908 | 10.1 |
| KE11242 | KT1108 | 2 | 11 | 111 | 11.11 |
| KE11242 | KT1621 | 1 | 11 | 111 | 11.11 |
| KE11648 | KT1108 | 3 | 11 | 111 | 11.11 |
| KE11648 | KT1621 | 3 | 11 | 111 | 11.11 |
| KE10692 | KT1441 | 12 | 2 | 415 | 2.4 |
| KE10742 | KT1359 | 17 | 2 | 415 | 2.4 |
| KE10742 | KT1441 | 6 | 2 | 415 | 2.4 |
| KE10937 | KT1359 | 19 | 2 | 415 | 2.4 |
| KE10937 | KT1441 | 8 | 2 | 415 | 2.4 |
| KE11087 | KT1445 | 5 | 2 | 415 | 2.6 |
| KE11149 | KT1445 | 7 | 2 | 415 | 2.6 |
| KE10167 | CL18 | 15 | 2 | 415 | A |
| KE10167 | KT1315 | 7 | 2 | 415 | A |
| KE10167 | KT1317 | 8 | 2 | 415 | A |
| KE10167 | KT1624 | 11 | 2 | 415 | A |
| KE10167 | KT1627 | 8 | 2 | 415 | A |
| KE11157 | CL18 | 10 | 2 | 415 | A |

| | | | | | |
|---|---|---|---|---|---|
| KE11157 | KT1315 | 10 | 2 | 415 | A |
| KE11157 | KT1317 | 15 | 2 | 415 | A |
| KE11157 | KT1624 | 19 | 2 | 415 | A |
| KE11157 | KT1627 | 14 | 2 | 415 | A |
| KE11218 | CL18 | 11 | 2 | 415 | A |
| KE11218 | KT1315 | 5 | 2 | 415 | A |
| KE11218 | KT1317 | 5 | 2 | 415 | A |
| KE11218 | KT1624 | 9 | 2 | 415 | A |
| KE11218 | KT1627 | 6 | 2 | 415 | A |
| KE11224 | CL18 | 14 | 2 | 415 | A |
| KE11224 | KT1315 | 11 | 2 | 415 | A |
| KE11224 | KT1317 | 9 | 2 | 415 | A |
| KE11224 | KT1624 | 9 | 2 | 415 | A |
| KE11224 | KT1627 | 7 | 2 | 415 | A |
| KE11280 | CL18 | 16 | 2 | 415 | A |
| KE11280 | KT1315 | 8 | 2 | 415 | A |
| KE11280 | KT1317 | 12 | 2 | 415 | A |
| KE11280 | KT1624 | 14 | 2 | 415 | A |
| KE11280 | KT1627 | 14 | 2 | 415 | A |
| KE11351 | CL18 | 14 | 2 | 415 | A |
| KE11351 | KT1315 | 9 | 2 | 415 | A |
| KE11351 | KT1317 | 10 | 2 | 415 | A |
| KE11351 | KT1624 | 4 | 2 | 415 | A |
| KE11351 | KT1627 | 3 | 2 | 415 | A |
| KE11368 | CL18 | 18 | 2 | 415 | A |
| KE11368 | KT1315 | 8 | 2 | 415 | A |
| KE11368 | KT1317 | 11 | 2 | 415 | A |
| KE11368 | KT1624 | 6 | 2 | 415 | A |
| KE11368 | KT1627 | 5 | 2 | 415 | A |
| KE11455 | CL18 | 15 | 2 | 415 | A |
| KE11455 | KT1315 | 8 | 2 | 415 | A |
| KE11455 | KT1317 | 10 | 2 | 415 | A |
| KE11455 | KT1624 | 14 | 2 | 415 | A |

| | | | | | |
|---|---|---|---|---|---|
| KE11455 | KT1627 | 9 | 2 | 415 | A |
| KE11457 | CL18 | 16 | 2 | 415 | A |
| KE11457 | KT1315 | 5 | 2 | 415 | A |
| KE11457 | KT1317 | 8 | 2 | 415 | A |
| KE11457 | KT1624 | 9 | 2 | 415 | A |
| KE11457 | KT1627 | 9 | 2 | 415 | A |
| KE11719 | CL18 | 16 | 2 | 415 | A |
| KE11719 | KT1315 | 7 | 2 | 415 | A |
| KE11719 | KT1317 | 10 | 2 | 415 | A |
| KE11719 | KT1624 | 11 | 2 | 415 | A |
| KE11719 | KT1627 | 10 | 2 | 415 | A |
| KE11771 | CL18 | 17 | 2 | 415 | A |
| KE11771 | KT1315 | 8 | 2 | 415 | A |
| KE11771 | KT1317 | 14 | 2 | 415 | A |
| KE11771 | KT1624 | 18 | 2 | 415 | A |
| KE11771 | KT1627 | 17 | 2 | 415 | A |
| KE11922 | CL18 | 13 | 2 | 415 | A |
| KE11922 | KT1315 | 9 | 2 | 415 | A |
| KE11922 | KT1317 | 12 | 2 | 415 | A |
| KE11922 | KT1624 | 11 | 2 | 415 | A |
| KE11922 | KT1627 | 8 | 2 | 415 | A |
| KE9775 | CL18 | 12 | 2 | 415 | A |
| KE9775 | KT1315 | 5 | 2 | 415 | A |
| KE9775 | KT1317 | 4 | 2 | 415 | A |
| KE9775 | KT1624 | 8 | 2 | 415 | A |
| KE9775 | KT1627 | 7 | 2 | 415 | A |
| KE10303 | KT1324 | 20 | 1 | 22 | C |
| KE10314 | KT1324 | 20 | 1 | 22 | C |
| KE11782 | KT1623 | 11 | 1 | 22 | D |
| KE11837 | KT1623 | 14 | 1 | 22 | D |
| KE9784 | KT1073 | 19 | 1 | 22 | D |
| KE9784 | KT1623 | 18 | 1 | 22 | D |
| KE10263 | KT1320 | 8 | 3 | 112 | E |

| | | | | | |
|---|---|---|---|---|---|
| KE10263 | KT1443 | 6 | 3 | 112 | E |
| KE10782 | KT1320 | 9 | 3 | 112 | E |
| KE10782 | KT1443 | 10 | 3 | 112 | E |
| KE10995 | KT1320 | 8 | 3 | 112 | E |
| KE10995 | KT1443 | 7 | 3 | 112 | E |
| KE11060 | KT1320 | 8 | 3 | 112 | E |
| KE11060 | KT1443 | 8 | 3 | 112 | E |
| KE11320 | KT1320 | 9 | 3 | 112 | E |
| KE11320 | KT1443 | 4 | 3 | 112 | E |
| KE10941 | KT1100 | 4 | 1 | 22 | G |
| KE10941 | KT1446 | 15 | 1 | 22 | G |
| KE11321 | KT1100 | 1 | 1 | 22 | G |
| KE11321 | KT1446 | 14 | 1 | 22 | G |
| KE11474 | KT1100 | 9 | 1 | 22 | G |
| KE11474 | KT1446 | 17 | 1 | 22 | G |
| KE9961 | KT1100 | 11 | 1 | 22 | G |
| KE9961 | KT1446 | 10 | 1 | 22 | G |
| KE9378 | KT1315 | 16 | 2 | 415 | H |
| KE9378 | KT1317 | 12 | 2 | 415 | H |
| KE9378 | KT1624 | 20 | 2 | 415 | H |
| KE9378 | KT1627 | 19 | 2 | 415 | H |
| KE10091 | KT1317 | 19 | 2 | 415 | Singleton |
| KE10490 | KT1324 | 4 | 1 | 22 | Singleton |
| KE10793 | KT1318 | 11 | 10 | 908 | Singleton |
| KE10793 | KT1323 | 10 | 10 | 908 | Singleton |
| KE11553 | KT1367 | 20 | 1 | - | Singleton |
| KE11582 | KT1346 | 1 | 1 | 22 | Singleton |
| KE11774 | KT1107 | 18 | 18 | 590 | Singleton |
