# Supplementary material for: Rising incidence of carbapenem-resistant Citrobacter spp. in a German tertiary-care hospital: epidemiology, clinical impact, and the role of the hospital wastewater system—findings from a six-year molecular study
Source: Microbiol Spectr. 2026 Jan 22;14(3):e02670-25. doi: 10.1128/spectrum.02670-25 (PMC12955474; doi:10.1128/spectrum.02670-25)
Supplement: File S5 — Room occupancy on ward X2. [file spectrum.02670-25-s0005.pdf]

Supplemental File 5: Room occupancy on ward X2

Subcluster	Patient	Stay on ward X2 in room 7	Stay on ward X2 in room 8
**E**	1E	-	-
	2E	02/15/22 to 02/18/22	08/29/22 to 09/01/22
	3E	01/10/23 to 01/20/23<br>01/23/23 to 01/31/23<br>02/24/23 to 03/10/23	11/08/22 to 01/09/23<br>08/08/23 to 08/10/23
	4E	-	03/09/23 to 03/22/23
	5E	-	10/27/23 to 11/15/23

Subcluster	Patient	Stay on ward X2 in room 3	Stay on ward X2 in room 5
**g**	1g	06/29/22 to 07/04/22	06/28/22 to 06/29/22
	2g	11/28/22 to 12/01/22	08/05/22 to 08/12/22
	3g	-	12/06/22 to 12/09/22

Subcluster	Patient	Stay on ward X2 in room 10
**G**	1G	-
	2G	-
	3G	-
	4G	-

Subcluster	Patient	Stay on ward X2 in room 10
**h**	1h	04/10/23 to 04/20/23
	2h	-
