# Supplementary material for: Rising incidence of carbapenem-resistant Citrobacter spp. in a German tertiary-care hospital: epidemiology, clinical impact, and the role of the hospital wastewater system—findings from a six-year molecular study
Source: Microbiol Spectr. 2026 Jan 22;14(3):e02670-25. doi: 10.1128/spectrum.02670-25 (PMC12955474; doi:10.1128/spectrum.02670-25)
Supplement: File S6 — Patient room occupancy. [file spectrum.02670-25-s0006.pdf]

Supplemental File 6: Concordance between patient room occupancy and environmental isolate recovery sites

Subcluster	Ward	Rooms in which subcluster isolates were detected	Patient	Patient room occupancy
**A**	X1	7 and 13	A3	Room 13 from 07/18/22 to 07/22/22
	X3	1, 4 and 6	A3	Room 4 from 08/15/23 to 08/22/23<br>Room 6 from 02/01/24 to 02/29/24
			A6	Room 4 from 09/16/23 to 09/20/23
			A11	Room 4 from 09/19/23 to 09/20/23<br>Room 6 from 08/02/24 to 08/24/24
**D**	X1	1 and 6	D4	Room 6 from 09/12/24 to 09/18/24
**E**	X2	7 and 8	2E	Room 7 from 02/15/22 to 02/18/22<br>Room 8 from 08/29/22 to 09/01/22
			3E	Room 7 from 01/10/23 to 01/20/23, 01/23/23 to 01/31/23 and 02/24/23 to 03/10/23<br>Room 8 from 11/08/22 to 01/09/23 and 08/08/23 to 08/10/23
			4E	Room 8 from 03/09/23 to 03/22/23
			5E	Room 8 from 10/27/23 to 11/15/23
**G**	X1	13	-	-
	X2	10	-	-

Supplemental File 6: Concordance between patient room occupancy and environmental isolate recovery sites

**a**	X1	1 and 5	1a	Room 1 from 10/10/20 to 10/29/20<br>Room 5 from 10/30/20 to 12/30/20
			3a	Room 1 from 07/31/24 to 09/09/24
**d**	X1	2 and 3	-	-
**e**	X3	8	2e	Room 8 from 01/15/24 to 01/16/24
**f**	X1	1 and 14	1f	Room 1 from 08/14/23 to 08/29/23
			2f	Room 1 from 06/24/24 to 06/26/24
**g**	X2	3 and 5	1g	Room 3 from 06/28/22 to 07/04/22
			2g	Room 3 from 07/25/22 to 08/01/22 and 02/12/22 to 03/12/22<br>Room 5 from 07/25/22 to 08/01/22
			3g	Room 5 from 12/06/22 to 12/09/22
**h**	X2	10	1h	Room 10 from 04/10/23 to 04/20/23
