# Supplementary material for: Rising incidence of carbapenem-resistant Citrobacter spp. in a German tertiary-care hospital: epidemiology, clinical impact, and the role of the hospital wastewater system—findings from a six-year molecular study
Source: Microbiol Spectr. 2026 Jan 22;14(3):e02670-25. doi: 10.1128/spectrum.02670-25 (PMC12955474; doi:10.1128/spectrum.02670-25)
Supplement: File S7 — Accession numbers. [file spectrum.02670-25-s0007.pdf]

Supplemental File 7:

Accession numbers of all patient and environmental strains included in the study

Accession No.: Unique, permanent identifier assigned to a specific sequence record in NCBI gene bank
SPUID: Submitter-Provided Unique ID
Organism: Species designation
Tax ID: Unique numeric identifier for a taxon in the NCBI Taxonomy database
ST: Sequence type of each isolate

Accession No.	SPUID	Organism	Tax ID	ST
SAMN50440649	KE10017	Citrobacter freundii	546	ST22
SAMN50440650	KE10036	Citrobacter freundii	546	ST908
SAMN50440651	KE10044	Citrobacter freundii	546	ST22
SAMN50440652	KE10055	Citrobacter freundii	546	ST272
SAMN50440653	KE10071	Citrobacter freundii	546	ST112
SAMN50440654	KE10091	Citrobacter freundii	546	ST415
SAMN50440655	KE10099	Citrobacter freundii	546	ST928
SAMN50440656	KE10115	Citrobacter freundii	546	ST98
SAMN50440657	KE10138	Citrobacter freundii	546	ST98
SAMN50440658	KE10151	Citrobacter freundii	546	ST95
SAMN50440659	KE10167	Citrobacter freundii	546	ST415
SAMN50440660	KE10186	Citrobacter freundii	546	ST702
SAMN50440661	KE10204	Citrobacter freundii	546	ST98
SAMN50440662	KE10205	Citrobacter freundii	546	ST19
SAMN50440663	KE10217	Citrobacter freundii	546	ST98
SAMN50440664	KE10233	Citrobacter freundii	546	ST98
SAMN50440665	KE10263	Citrobacter freundii	546	ST112
SAMN50440666	KE10286	Citrobacter freundii	546	ST22
SAMN50440667	KE10303	Citrobacter freundii	546	ST22
SAMN50440668	KE10314	Citrobacter freundii	546	ST22
SAMN50440669	KE10388	Citrobacter freundii	546	ST153
SAMN50440670	KE10444	Citrobacter freundii	546	ST22
SAMN50440671	KE10451	Citrobacter freundii	546	ST22
SAMN50440672	KE10459	Citrobacter freundii	546	ST22
SAMN50440673	KE10490	Citrobacter freundii	546	ST22
SAMN50440674	KE10504	Citrobacter freundii	546	ST22
SAMN50440675	KE10581	Citrobacter freundii	546	ST415
SAMN50440676	KE10589	Citrobacter freundii	546	ST18
SAMN50440677	KE10692	Citrobacter freundii	546	ST415
SAMN50440678	KE10724	Citrobacter freundii	546	ST18
SAMN50440679	KE10742	Citrobacter freundii	546	ST415
SAMN50440680	KE10748	Citrobacter freundii	546	ST150
SAMN50440681	KE10750	Citrobacter freundii	546	ST257
SAMN50440682	KE10755	Citrobacter freundii	546	ST19
SAMN50440683	KE10782	Citrobacter freundii	546	ST112
SAMN50440684	KE10793	Citrobacter freundii	546	ST908
SAMN50440685	KE10821	Citrobacter freundii	546	ST112
SAMN50440686	KE10867	Citrobacter freundii	546	ST508
SAMN50440687	KE10916	Citrobacter freundii	546	ST415

SAMN50440688	KE10937	Citrobacter freundii	546	ST415
SAMN50440689	KE10941	Citrobacter freundii	546	ST22
SAMN50440690	KE10994	Citrobacter freundii	546	ST928
SAMN50440691	KE10995	Citrobacter freundii	546	ST112
SAMN50440692	KE11015	Citrobacter freundii	546	ST508
SAMN50440693	KE11038	Citrobacter freundii	546	ST257
SAMN50440694	KE11059	Citrobacter freundii	546	ST508
SAMN50440695	KE11060	Citrobacter freundii	546	ST112
SAMN50440696	KE11066	Citrobacter freundii	546	ST22
SAMN50440697	KE11079	Citrobacter freundii	546	ST257
SAMN50440698	KE11087	Citrobacter freundii	546	ST415
SAMN50440699	KE11116	Citrobacter freundii	546	ST112
SAMN50440700	KE11149	Citrobacter freundii	546	ST415
SAMN50440701	KE11157	Citrobacter freundii	546	ST415
SAMN50440702	KE11164	Citrobacter freundii	546	ST91
SAMN50440703	KE11166	Citrobacter freundii	546	ST415
SAMN50440704	KE11195	Citrobacter freundii	546	ST64
SAMN50440705	KE11197	Citrobacter freundii	546	ST112
SAMN50440706	KE11218	Citrobacter freundii	546	ST415
SAMN50440707	KE11224	Citrobacter freundii	546	ST415
SAMN50440708	KE11227	Citrobacter freundii	546	no ST
SAMN50440709	KE11242	Citrobacter freundii	546	ST111
SAMN50440710	KE11244	Citrobacter freundii	546	ST22
SAMN50440711	KE11254	Citrobacter freundii	546	ST580
SAMN50440712	KE11280	Citrobacter freundii	546	ST415
SAMN50440713	KE11296	Citrobacter freundii	546	ST22
SAMN50440714	KE11297	Citrobacter freundii	546	ST257
SAMN50440715	KE11320	Citrobacter freundii	546	ST112
SAMN50440716	KE11321	Citrobacter freundii	546	ST22
SAMN50440717	KE11351	Citrobacter freundii	546	ST415
SAMN50440718	KE11368	Citrobacter freundii	546	ST415
SAMN50440719	KE11389	Citrobacter freundii	546	ST112
SAMN50440720	KE11391	Citrobacter freundii	546	ST22
SAMN50440721	KE11414	Citrobacter freundii	546	ST22
SAMN50440722	KE11415	Citrobacter freundii	546	ST908
SAMN50440723	KE11455	Citrobacter freundii	546	ST415
SAMN50440724	KE11457	Citrobacter freundii	546	ST415
SAMN50440725	KE11474	Citrobacter freundii	546	ST22
SAMN50440726	KE11512	Citrobacter freundii	546	ST112
SAMN50440727	KE11545	Citrobacter freundii	546	ST22
SAMN50440728	KE11546	Citrobacter freundii	546	ST18
SAMN50440729	KE11553	Citrobacter freundii	546	no ST
SAMN50440730	KE11560	Citrobacter freundii	546	ST22
SAMN50440731	KE11568	Citrobacter freundii	546	ST216
SAMN50440732	KE11582	Citrobacter freundii	546	ST22
SAMN50440733	KE11603	Citrobacter freundii	546	ST112
SAMN50440734	KE11609	Citrobacter freundii	546	ST11

SAMN50440735	KE11620	Citrobacter freundii	546	ST8
SAMN50440736	KE11648	Citrobacter freundii	546	ST111
SAMN50440737	KE11689	Citrobacter freundii	546	ST22
SAMN50440738	KE11703	Citrobacter freundii	546	ST702
SAMN50440739	KE11719	Citrobacter freundii	546	ST415
SAMN50440740	KE11727	Citrobacter freundii	546	ST22
SAMN50440741	KE11728	Citrobacter freundii	546	ST257
SAMN50440742	KE11759	Citrobacter freundii	546	ST22
SAMN50440743	KE11761	Citrobacter freundii	546	ST11
SAMN50440744	KE11771	Citrobacter freundii	546	ST415
SAMN50440745	KE11774	Citrobacter freundii	546	ST590
SAMN50440746	KE11782	Citrobacter freundii	546	ST22
SAMN50440747	KE11789	Citrobacter freundii	546	ST112
SAMN50440748	KE11799	Citrobacter freundii	546	ST91
SAMN50440749	KE11807	Citrobacter freundii	546	ST145
SAMN50440750	KE11835	Citrobacter freundii	546	ST632
SAMN50440751	KE11837	Citrobacter freundii	546	ST22
SAMN50440752	KE11841	Citrobacter freundii	546	ST112
SAMN50440753	KE11856	Citrobacter freundii	546	ST18
SAMN50440754	KE11857	Citrobacter freundii	546	ST22
SAMN50440755	KE11863	Citrobacter freundii	546	ST11
SAMN50440756	KE11898	Citrobacter freundii	546	ST18
SAMN50440757	KE11904	Citrobacter freundii	546	ST18
SAMN50440758	KE11922	Citrobacter freundii	546	ST415
SAMN50440759	KE11940	Citrobacter freundii	546	ST18
SAMN50440760	KE9068	Citrobacter freundii	546	ST22
SAMN50440761	KE9314	Citrobacter freundii	546	ST22
SAMN50440762	KE9378	Citrobacter freundii	546	ST415
SAMN50440763	KE9511	Citrobacter freundii	546	ST415
SAMN50440764	KE9554	Citrobacter freundii	546	ST112
SAMN50440765	KE9559	Citrobacter freundii	546	ST62
SAMN50440766	KE9650	Citrobacter freundii	546	ST22
SAMN50440767	KE9724	Citrobacter freundii	546	ST18
SAMN50440768	KE9752	Citrobacter freundii	546	no ST
SAMN50440769	KE9775	Citrobacter freundii	546	ST415
SAMN50440770	KE9784	Citrobacter freundii	546	ST22
SAMN50440771	KE9790	Citrobacter freundii	546	ST22
SAMN50440772	KE9831	Citrobacter freundii	546	ST98
SAMN50440773	KE9890	Citrobacter freundii	546	ST22
SAMN50440774	KE9957	Citrobacter freundii	546	ST22
SAMN50440775	KE9961	Citrobacter freundii	546	ST22
SAMN50440776	KE9998	Citrobacter freundii	546	ST22
SAMN50440777	KE9999	Citrobacter freundii	546	ST415
SAMN50440778	KE11267	Citrobacter braakii	57706	no ST
SAMN50440779	KE11409	Citrobacter braakii	57706	ST225
SAMN50440780	KE10746	Citrobacter braakii	57706	ST268
SAMN50440781	KE11302	Citrobacter braakii	57706	ST281

SAMN50440782	KE10910	Citrobacter portucalensis	1639133	ST129
SAMN50440783	KE11464	Citrobacter portucalensis	1639133	no ST
SAMN50440784	KE9539	Citrobacter portucalensis	1639133	ST96
SAMN50440785	KE9960	Citrobacter portucalensis	1639133	ST129
SAMN50440786	KE11445	Citrobacter pasteurii	1563222	no ST
SAMN50440787	KT1073	Citrobacter freundii	546	ST22
SAMN50440788	CL18	Citrobacter freundii	546	ST415
SAMN50440789	CL6	Citrobacter freundii	546	ST908
SAMN50440790	KT1323	Citrobacter freundii	546	ST908
SAMN50440791	KT1325	Citrobacter freundii	546	no ST
SAMN50440792	KT1326	Citrobacter freundii	546	ST415
SAMN50440793	KT1441	Citrobacter freundii	546	ST415
SAMN50440794	KT1443	Citrobacter freundii	546	ST112
SAMN50440795	KT1094	Citrobacter freundii	546	ST22
SAMN50440796	KT1099	Citrobacter freundii	546	ST22
SAMN50440797	KT1100	Citrobacter freundii	546	ST22
SAMN50440798	KT1107	Citrobacter freundii	546	ST590
SAMN50440799	KT1108	Citrobacter freundii	546	ST111
SAMN50440800	KT1319	Citrobacter freundii	546	ST112
SAMN50440801	KT1320	Citrobacter freundii	546	ST112
SAMN50440802	KT1347	Citrobacter freundii	546	ST112
SAMN50440803	KT1348	Citrobacter freundii	546	ST112
SAMN50440804	KT1367	Citrobacter freundii	546	ST22
SAMN50440805	KT1358	Citrobacter freundii	546	ST415
SAMN50440806	KT1359	Citrobacter freundii	546	ST415
SAMN50440807	KT1360	Citrobacter freundii	546	ST415
SAMN50440808	KT1069	Citrobacter freundii	546	ST22
SAMN50440809	KT1324	Citrobacter freundii	546	ST22
SAMN50440810	KT1346	Citrobacter freundii	546	ST22
SAMN50440811	KT1446	Citrobacter freundii	546	ST22
SAMN50440812	KT1622	Citrobacter freundii	546	ST22
SAMN50440813	KT1623	Citrobacter freundii	546	ST22
SAMN50440814	KT1621	Citrobacter freundii	546	ST111
SAMN50440815	KT1315	Citrobacter freundii	546	ST415
SAMN50440816	KT1317	Citrobacter freundii	546	ST415
SAMN50440817	KT1344	Citrobacter freundii	546	ST415
SAMN50440818	KT1445	Citrobacter freundii	546	ST415
SAMN50440819	KT1624	Citrobacter freundii	546	ST415
SAMN50440820	KT1627	Citrobacter freundii	546	ST415
SAMN50440821	KT1318	Citrobacter freundii	546	ST908
SAMN50440822	KT1625	Citrobacter freundii	546	ST928
SAMN50440823	KT1444	Citrobacter freundii	546	no ST
